# 3D Spatially Resolved Models of the Intracellular Dynamics of the Hepatitis C Genome Replication Cycle

**DOI:** 10.3390/v9100282

**Published:** 2017-09-30

**Authors:** Markus M. Knodel, Sebastian Reiter, Paul Targett-Adams, Alfio Grillo, Eva Herrmann, Gabriel Wittum

**Affiliations:** 1 Dipartimento di Scienze Matematiche (DISMA) “G.L. Lagrange”, Politecnico di Torino, Corso Duca degli Abruzzi, 24, 10129 Torino (TO), Italy; alfio.grillo@polito.it; 2 Goethe Center for Scientific Computing (G-CSC), Goethe Universität Frankfurt, Kettenhofweg 139, 60325 Frankfurt am Main, Germany; sreiter@gcsc.uni-frankfurt.de (S.R.); wittum@gcsc.uni-frankfurt.de or gabriel.wittum@kaust.edu.sa (G.W.); 3 Applied Mathematics and Computational Science, Computer, Electrical and Mathematical Science and Engineering Division, King Abdullah University of Science and Technology, KAUST, Thuwal 23955, Saudi Arabia; 4 Medivir AB, Department of Biology, Huddinge 141 22, Sweden; Paul.Targett-Adams@medivir.com; 5 Department of Medicine, Institute for Biostatistics and Mathematic Modeling, Goethe Universität Frankfurt, Theodor-Stern-Kai 7, 60590 Frankfurt am Main, Germany; herrmann@med.uni-frankfurt.de

**Keywords:** computational virology, hepatitis C virus (HCV), viral dynamics, within-host viral modelling, mathematical models of viral RNA cycle, 3D spatio-temporal resolved mathematical models, realistic geometries, (surface) partial differential equations, Finite Volumes, massively parallel multigrid solvers

## Abstract

Mathematical models of virus dynamics have not previously acknowledged spatial resolution at the intracellular level despite substantial arguments that favor the consideration of intracellular spatial dependence. The replication of the hepatitis C virus (HCV) viral RNA (vRNA) occurs within special replication complexes formed from membranes derived from endoplasmatic reticulum (ER). These regions, termed membranous webs, are generated primarily through specific interactions between nonstructural virus-encoded proteins (NSPs) and host cellular factors. The NSPs are responsible for the replication of the vRNA and their movement is restricted to the ER surface. Therefore, in this study we developed fully spatio-temporal resolved models of the vRNA replication cycle of HCV. Our simulations are performed upon realistic reconstructed cell structures—namely the ER surface and the membranous webs—based on data derived from immunostained cells replicating HCV vRNA. We visualized 3D simulations that reproduced dynamics resulting from interplay of the different components of our models (vRNA, NSPs, and a host factor), and we present an evaluation of the concentrations for the components within different regions of the cell. Thus far, our model is restricted to an internal portion of a hepatocyte and is qualitative more than quantitative. For a quantitative adaption to complete cells, various additional parameters will have to be determined through further in vitro cell biology experiments, which can be stimulated by the results described in the present study.

## 1. Introduction

The hepatitis C virus (HCV) only infects human (and some higher primate) liver cells. Even though HCV-infected liver cells (hepatocytes) may initially remain viable, culminative stress to the liver following a period of about 10–20 years infection predisposes patients to develop liver cirrhosis and liver cancer. Liver damage caused by HCV infection is the main cause for liver transplantations in the Western World. Substantial efforts to understand the mechanisms underpinning HCV replication [1,2,3], have culminated in the the development of effective direct acting antivirals (DAAs) [4] against HCV, which now offer substantial cure rates for many patients infected with the virus.

However, many facets of HCV replication remain poorly understood. In particular, even though various central steps of virus genome replication could be revealed experimentally, still many processes are understood only in part and/or only qualitatively. A deeper insight into the exact molecular dynamics of HCV replication will enhance, for example, the understanding of the interplay between viral proteins, viral RNA (vRNA) replication, and cell metabolism. This knowledge will likely serve to enhance our understanding of the impact of direct antiviral agents (DAAs) upon the interplay of vRNA and viral proteins. Additionally, such understanding could be used to reveal global mechanisms involved in replication of related viruses.

Mathematical descriptions for the dynamics of biophysical processes have become better refined within the last years. In the case of virus dynamics, compartmental kinetic models based on ordinary differential equations (ODEs) now represent the state of the art [5,6,7,8,9,10,11,12,13,14,15,16,17,18]. In fact, such ODEs represent homogenized descriptions of processes for which the spatial resolution is not accounted for. For many processes, such homogenizations are justified and useful. (In this study, the term “homogenization” refers to the averaging of the dynamics over the complete space rather then other meanings which appear in other contexts.) Therefore, ODE-based models of HCV dynamics allowed for new and important insights. However, the investigation of those effects which depend on the spatial location of the components, requires a description based on partial differential equations (PDEs) that need to be solved on computational domains that reflect realistic geometric structures. PDEs, indeed, allow to describing effects such as the spatial distribution of the agents concurring to the evolution of the vRNA, that cannot be resolved otherwise, and permit to account for the influence of geometry on the spatio-temporal evolution of the unknowns. Realistic reconstructed geometries of cell structures are often the most appropriate basis for the in silico evaluation of spatially resolved models, since they reflect best the role of the real geometry upon the modeled processes, and influence that cannot be obtained by simplified geometric objects.

In the literature, there already exist models that describe virus dynamics with the aid of PDEs, cf. [19,20,21]. In [19], the authors elaborated the global stability for a class of diffusion equations which appear in computational biology. They studied the global stability of constant steady-state solutions for several reaction-diffusion systems with and without delay. The models harbored up to one dimension in space and inherited in part time delayed coefficients. The solution techniques were based upon a variational principle: The authors constructed Lyapunov functionals for PDEs and delayed partial differential equations (DPDEs) using Lyapunov functionals for ODEs and delayed differential equations (DDEs). They applied their framework to study the global stability of several models from virology and epidemiology. We mention in particular the consideration of 1D PDE model of [22] which described the reaction-diffusion system of infected and uninfected hepatocytes which were considered to be immobile, while viruses were consideder to move freely following the Fickian diffusion law. Moreover, the authors of [19] constructed a Lyapunov functional and studied the stability of the model of [22]. Indeed, the first diffusive model of [22] was an extension of former ODE models [23,24,25] to the spatial case. In detail, Wang and Wang [22] introduced a HBV model by means of spatial dependence in such a way that viruses move freely in the liver, but healthy and infected hepatocytes (liver cells) are immobile. Hence, the original model coupled all three concentrations by means of a coupled ODE-PDE system. This model was improved within various steps [26,27,28]. In [20], the authors generalized and extended all these HBV models by means of a time-delayed coupled ODE-PDE system, i.e., they presented a hepatitis B virus (HBV) model with spatial diffusion and time delays [20]. In particular, Hattaf and Yousfi [20] extended and generalized various diffusive HBV models of the literature, which are based upon the first approach of [22]. Lyapunov functionals were constructed for the analysis of the equilibrium in the cases of disease-free and chronic infection. Hattaf and Yousfi [20] also performed the important intensive theoretical investigation for proving that there exists a unique solution of their basic equation system and that this solution remained non-negative and bounded. Constructing Lyapunov functionals, the authors investigated in detail the asymptotic stability of the disease-free case and of the chronic infection equilibrium case and derived distinct conditions for this stability. The results of the theoretical investigations were applied to the extended model which harbored homogeneous Neumann boundary conditions. They investigated the 1D system using various different parameters to test for the sensitivity of the results to the diffusion coefficient and to the time delays. They concluded that the diffusion coefficient caused different virus loads at different spatial locations and, therefore, the time scale to arrive at the chronic infection equilibrium depended upon the diffusion coefficient of the virus, even though in their case the virus diffusion showed no effect on the asymptotic properties of the equilibrium. This fits to the assumption of a unique medium where the processes take place. The results of [20] demonstrated the importance to incorporate spatial resolution within computational virology. In [21], the authors introduced a numerical method for coupled DPDEs which allowed for the evaluation of viral infection models similar to the previously described HBV work [20], but now extended to HIV. The paper presented a model of 3 coupled equations which described similar biology to [20]: Two of the equations are DDEs and one is a DPDE. (Similar to the case before, the model describes the reactive and diffusive properties of the dynamics of the densities of uninfected cells, infected cells and free virus at a spatial position and time, respectively.) The focus of that work was the introduction of numerically stable methods to solve such equation systems. The authors introduce Finite Difference (FD) schemes for the temporal and spatial derivations. They proved that the solutions of the discretized version of their delayed discrete model remained nonnegative and bounded, hence the numerical method could be applied safely. They investigated the global stability of the DPDEs system by constructing discrete Lyapunov functionals and proved the asymptotically stability of the system for the disease-free case and for the chronic infection case. They further demonstrated that the proposed method preserved the global stability of equilibria of the continuous system with no restriction on the space and time step sizes. The authors derived parameter conditions for the cases when the disease-free equilibrium was globally stable which, biologically-speaking, gave conditions for when the infection was resolved compared to chronic.

In summary, at the scale of the virus-infected patient, there exist models of the spatially resolved dynamics of different viruses corresponding to the interplay of the uninfected cells, infected cells, and free virus concentrations [19,20,21] as discussed previously. The techniques corresponding to the author’s PDEs and delayed PDEs (DPDEs) are based upon Lyapunov functionals and the models are extensions of former ODE models [23,24,25] to the spatially resolved case. As discussed previously, these models and methods allowed for interesting insight at the scale of the complete liver.

However, to the best of our knowledge, studies performing such analyses at the (intra)cellular scale have not been published yet. Therefore, our aim was to perform a similar approach to [19,20,21], but at a (intra)cellular level. We remark that in opposite to the scale of the complete liver where single non-distinguishable hepatocytes form the basis of the medium, the inner structure of single hepatocytes shows significant non-continuous structure, hence the effects originating from the medium structure may be expected to be even stronger compared to the scale of the complete liver.

There is strong biological evidence that the spatial dependence is a crucial factor for the vRNA replication of HCV at a (intra)cellular level [2,29]. Such replication takes place within specialized compartments of virus-infected hepatocytes, which form from the surface of the Endoplasmatic Reticulum (ER) [1,2]. The subsequent remodelling of the ER membrane by HCV results in a structure termed the “membranous web”, which houses replication complexes that serve as factories for vRNA replication and bridge the virus-dependent process of genome replication and packaging. It has proven to be difficult to experimentally capture the formation of the membranous web [30] and the trafficking of its components, namely host and viral proteins and vRNA. Trafficking of the components take place within the complex three dimensional space, which is determined by the shape of the modified ER membranes that serve to form the membranous web. An important example that demonstrated the requirement for spatial dependence of HCV components is highlighted through the behaviour of the HCV-encoded NSP NS5A in response to specific pharmacological-mediated inhibition by NS5A inhibitors [31,32]. Such small molecules cause a spatial redistribution of the viral protein and change its intracellular mobility patterns [33].

Therefore, multiple levels of rationale exist for developing computational models that resolve HCV replication dynamics both in time (as ODE models do), and in space (as PDEs allow for).

HCV virus replication is based upon two basic processes [1,3,18]: The vRNA gets replicated (vRNA cycle) and then combined (packaged) with structural virus proteins (SPs) to form new progeny virions (virus assembly).

The present study introduces relatively simple but fully 3D spatio-temporal resolved models of the interplay between vRNA, nonstructural viral proteins (NSP), membranous web (web) and a generic host factor (host) in order to recapitulate the basic properties of the vRNA replication cycle. The models are evaluated on a realistic geometric intracellular environment, based upon reconstructed ER surface and membranous web regions from experimentally-derived imaging data. On top of this geometric setup, we developed and implemented the models we present here.

We describe the replication cycle of the viral genome (vRNA) by means of a set of nonlinear coupled partial differential equations (PDEs). These equations are discretized by means of a Finite Volume method (FV). The arising equations are solved by employing massively parallel multigrid solvers [34]. The computations are performed with the aid of the simulation software UG4 [34,35,36] on leading supercomputers [37].

Even though there exist already advanced quantitative data of HCV replication dynamics averaged over the entire cell [38,39], it is rather difficult to get exact quantitative data of the spatio-temporal behaviour of all the components of the vRNA replication cycle. Recently, we have estimated the diffusion constant of the NS5A protein of HCV. (The technical basics of the estimation are already published [37,40] while the parameter estimation itself based on experimental FRAP time series has not been published yet). In the present study we use heuristic values for the parameters of a new diffusion-reaction model and we restrict its evaluation to a specific (intra)cellular localisation of a realistic reconstructed cell.

Moving forward, detailed experimental setups can be developed that allow for estimating the necessary additional parameters required to develop the model further. This interdisciplinary interaction will hopefully catalyze more detailed insights into HCV replication properties. Further, approaches such as the one described by the current study may also form the basis for spatially resolved models of other viral infections.

This paper is organized as follows: First, we describe the reconstruction process of the ER surface and the web regions, and we introduce surface and volume meshes of the computational domains. Then, we introduce two spatial models, which both capture important aspects of the spatial behavior of the basic components of the vRNA cycle, i.e., a surface PDE (sPDE) model and a PDE model evaluated in the complete volume of the cell, rather than on the surface of the compartments. The sPDE model captures the fact that the NSPs anchor to the ER surface directly after their cleavage [1], i.e., that viral nonstructural protein movement is restricted to the ER surface. Indeed, using mathematical language, the ER surface can be considered to be a curved 2D tubular manifold embedded in the three-dimensional Euclidian space. Moreover, we also introduce a “normal” PDE model within the volume, which takes into account the fact that, presumably, the other components of the vRNA cycle (like the vRNA itself or the host factor) may move within the cytosol.

In future research, we intend to combine these two approaches. Here, we present simulations and evaluations for both models separately. At the end, we discuss our results and put them into the context of current and future research.

## 2. Materials and Methods

### 2.1. Biological Basis: Viral Genome Replication Cycle

The Hepatitis C virus belongs to the Flaviviridae family of the so-called plus stranded RNA viruses [3]. The central property of such viruses is that they heavily parasitise the host cell machinery in order effect their own replicative cycle: The vRNA, once released inside the cell from its protective protein shell (termed the capsid), docks directly at the ribosomes, which synthesize the viral polyproteins. The polyprotenis ase subsequently cleaved by host- and virus-proteases. The resulting non structural proteins (NSP) function to replicate the viral genome, while the structural proteins (SPs) serve to encapsidate the newly replicated vRNA to form progeny virions that are released from the cell with the aim of infecting other cells. The ER lumen does not function directly within the vRNA replication cycle. For details of the HCV replication cycle, we refer to [1,3].

The models we present here reproduce the central elements of the HCV vRNA reproduction cycle: vRNA diffuses away from its initial location where it was released from the virus particle and attaches to ribosomes. At the ribosomes, NSPs are produced and diffuse away away to form membranous webs at ER surfaces. Then, vRNA diffuses to the membranous webs and is replicated. The new vRNA diffuses to the ribosomes where the same cycle starts again. Since we use a reconstructed ER geometry and reconstructed regions of the membranous webs based on experimental data from immunostained hepatocyte-derived tissue culture cells, the ER surface and the regions where the membranous webs grow correspond to their respective locations within the used experimental data.

**Figure 1:**
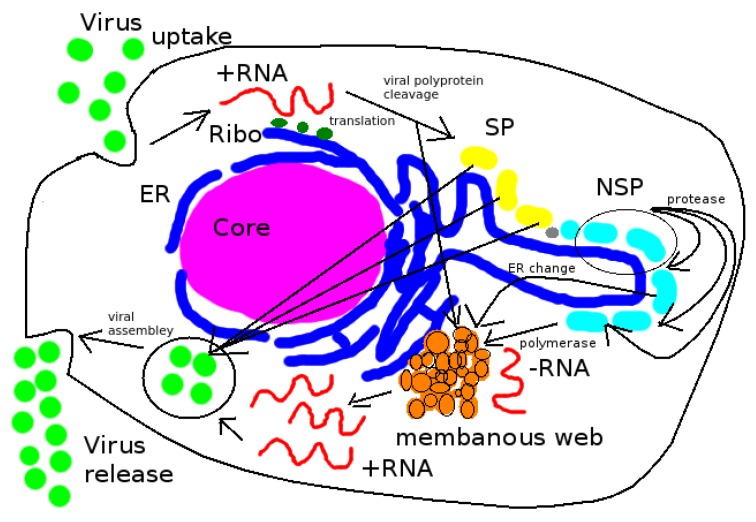
The HCV viral replication cycle: Virus endocytosis, vRNA uncoating, vRNA translates polyprotein, polyprotein splitting into structural and nonstructural proteins (SP/NSP), NSPs create membranous web at ER, vRNA replication inside web, new vRNA and SPs assembled to new virus particles, exocytosis of complete new viruses which infect other cells.

We emphasize that, as a first approach, we assume all NSPs to act together. Therefore, we use just one NSP and omit details of cleavage into various NSPs and SPs. (Indeed, there exist various NSPs like NS3, NS4a, NS4b, NS5a, NS5b). Thus, we only consider the state of polyprotein production, cleavage and accumulation of the NSPs at the webs. Further, since we only consider the vRNA cycle, we consider only nonstructural viral protein production. Structural proteins are not taken into account, but could be added within model extensions. Figure 1 depicts a rough overview scheme of the HCV replication cycle.

### 2.2. The Realistic Reconstructed Geometry

We used 3D confocal microscopy data of infected hepatocytes [29]. The ER and the membraneous web were labelled with specific markers (calnexin and dsRNA, respectively) which served as identifiers for surface reconstruction of the ER and the membraneous web regions. Using this experimental data, we reconstructed surfaces of various ERs and we reconstructed the regions of the membranous webs inside such cells [40].

#### 2.2.1. The Surface Reconstruction

**Figure 2:**
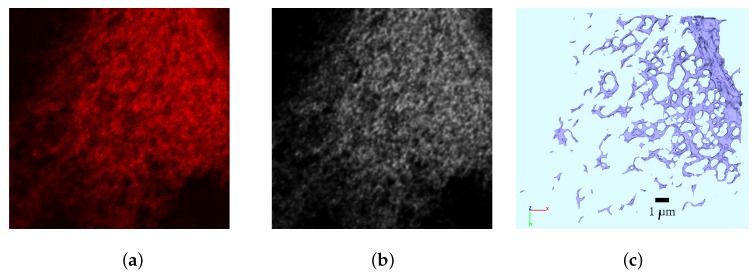
Reconstruction of ER surface (channel of cell data stained with calnexin)—special example: single steps of reconstruction process: (**a**) Raw confocal z-stacks, Calnexin ER-marker (**b**) deblured based on Huygens SVI (**c**) Surface mesh of (with NeuRA2) reconstructed ER surface.

The surface reconstruction process was based on the separate channels of the image stacks (deblurred with the aid of Huygens SVI [41]) and performed as follows: First, the surfaces were shaped with the aid of a specialized inertia momentum based anisotropic filter [42,43], which originally was developed for the reconstruction of highly noisy data in neuroscience but which applied perfectly also to the highly noisy stacks of the stained hepatocytes. Then the segmentation was performed by using advanced segmentation methods, namely the Hysteresis and Otsu [44] segmentation. Finally, a triangular surface mesh was created by means of the application of the marching cubes algorithm [45,46]. All steps (besides the deblurring) were performed with the aid of NeuRA2 [40,42,43] (NeuRA2 version 3 (NeuRA2.3), Steinbeis Research Institute Simulation in Technology, Ölbronn-Dürrn, Germany). For more details concerning the technical aspects of the reconstructions of the ER surface and the membranous webs, we refer to [40]. Post processing of the surface grid was performed with the aid of ProMesh [47]. We clarify that, in this context, post processing means to improve the quality of the triangles so that the computational mesh is adequate for simulation computations. The post processing does not appreciably change the shape of the surface any more, although some small local changes are possible due to the improvement of the quality of the triangles, but these changes are much smaller than the experimental error of the imaging process. The major steps of the reconstruction process are depicted in Figure 2.

### 2.3. The Surface Mesh for the Model Development

**Figure 3:**
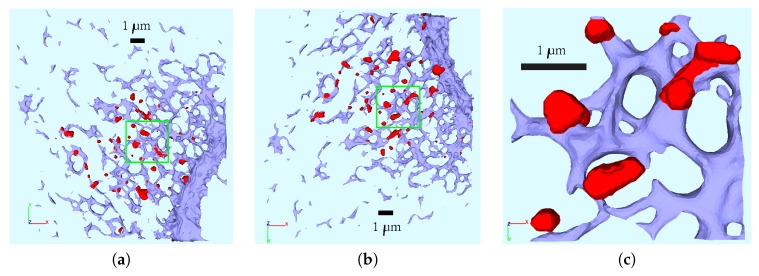
Reconstructed ER surface and web regions—choice of the cutout region for the 3D spatio-temporal resolved model development. Surface of ER (blue) and membranous web (red). Green frame marks for cutout choice. (**a**) Front view of complete cell (**b**) Back view of complete cell (**c**) Small cutout part of ER (E in blue, W web: in red).

For the sake of simplicity, we started to develop our spatially resolved models on a (intra)cellular portion of one of the reconstructed cells rather than complete cells. An extension to complete cells is a focus for subsequent work. Figure 3 depicts that reconstructed cell which we have chosen as basis for our development of 3D spatio-temporal resolved models of the vRNA replication cycle. In fact, Figure 3 shows the ER surface and the reconstructed surfaces of the membranous webs. In particular, in this figure, we demonstrate the choice of the specific intracellular region for our model development, cf., Figure 3c.

The window in which the cutout of the ER and the web regions are reported has dimensions 3.45 μm (length) × 3.36 μm (height) × 0.87 μm (depth).

**Figure 4:**
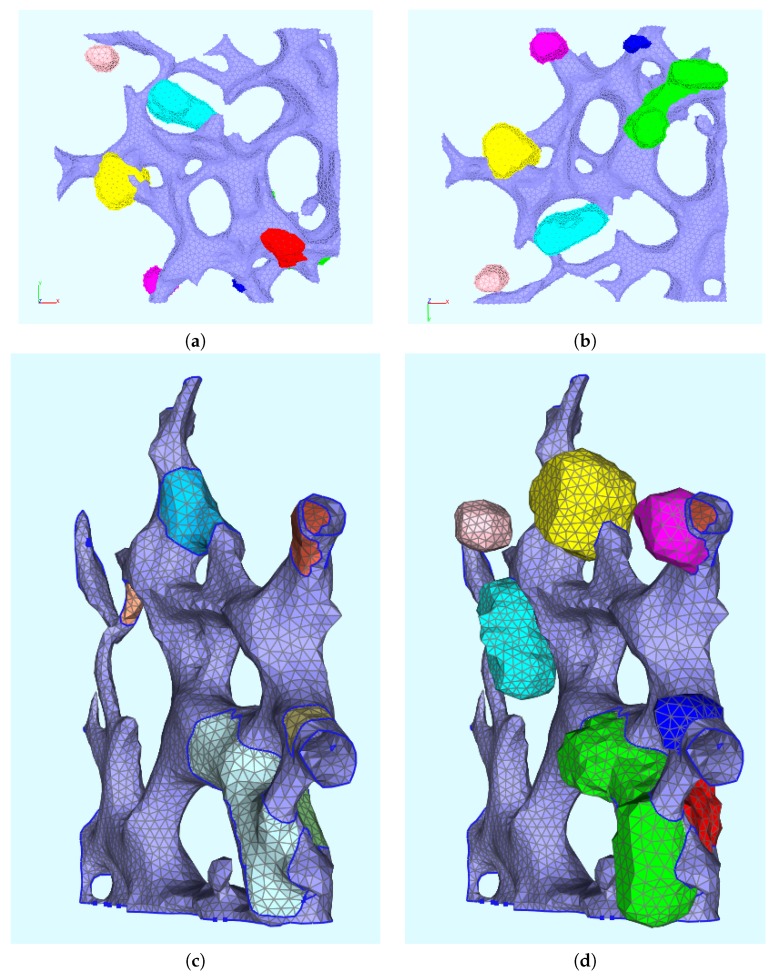
Subdomains of the small geometry for the development of the surface model. (**a**,**b**) Front view and back view; (**c**,**d**) ER rotated slightly; (**c**) webs unvisible, ribosomes open; (**d**) webs visible, ribosomes hidden. All surfaces together form the computation domain D. Middle blue: E, other colors: single web regions $W_{i}$ and ribosomal regions $R_{i}$, i=1,2,…7.

The surface domain D consists out of two regions: The triangulated ER surface, ℰ, and the trianglulated membranous web surfaces, 𝒲. Hence, the starting point of the geometric setup are the reconstructed surface of the ER and of the web regions of a cutout part of a real cell. We let ℰ represent the surface of the ER, and 𝒲 the union of all surfaces of the reconstructed web regions. Hence, D=E∪W is the surface obtained by the union of the surface of the ER, E, and the union of the surfaces of the (eight) unconnected web regions, $W=\cup_{i=1}^{7}W_{i}$. In Figure 3c, the web regions $W_{i}$, i=1,2,…,7, are marked in red. Each web surface, $W_{i}$, i=1,2,…,7, and the ER surface, E, define a subdomain. The intersection $R_{i}=E\cap W_{i}$ is a subdomain for each i=1,2,…,7 and we consider these intersections to be ribosomal regions. This means that we assume that the ribosomes are located at the interface of the ER surface and those sites where the webs are growing. The assumption that the ribosomes are located at the interface of ER surface and the webs growing on top of the ER surface is motivated by the fact that the ribosomes are located at special sites of the ER surface and that the translation of the viral proteins has to take place at the ribosomes. We assume that the polyprotein cleavage and the web growth (accumulation of NSPs) occur directly there, where the polyprotein is translated. We consider the ribosomal subdomain union to $R=\cup_{i=1}^{7}R_{i}$. The single subdomains of the computational domain are depicted in Figure 4 from different perspectives with pairwise different colors.

In the sequence, the surface grid will be used as a basis for the development of the surface partial differential equation (sPDE) models of the vRNA replication cycle.

#### 2.3.1. The Volume Mesh

This surface domain D was embedded into a rectangular hexahedron and the inner space was meshed with a tetrahedral grid. Thus, we embed D into a rectangular hexahedron, whose inner space we mesh with a tetrahedral volume grid, generated with the aid of TetGen [48] (Version 1.5, Research Group Numerical Mathematics and Scientific Computing, Weierstrass Institute for Applied Analysis and Stochastics (WIAS), Berlin, Germany).

**Figure 5:**
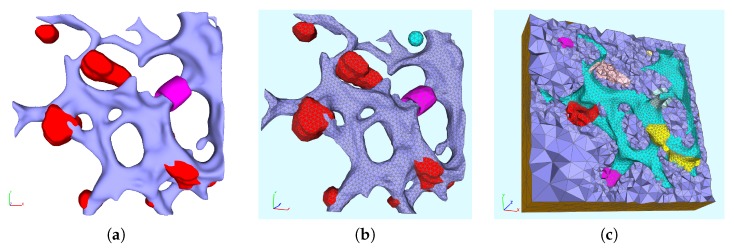
**(a)**: ER geometry of the cutout enriched by the ribosomic belt. **(b)**: Surface grid enriched by the sphere where the RNA start concentration will be located. **(c)**: Clip plane of the tetrahedralized domain Ω. Subdomains of the tetrahedral volume element, i.e., part of the cell where the ER lumen is excluded. Subdomains: cytosol dark blue, ER surface cyan, the colored “blobs” are the web subdomains. The complete region Ω represents the computational domain.

This discretization is such that the outermost elements of the volume grid share one of their sides with those of the pre-existing surface grid covering D. Hence, E and W represent internal boundaries among the volume regions of lumen, cytosol $C_{V}$ and web regions $W_{V}$. Moreover, Ω is the volume grid surrounding the ER surface E (cf., Figure 5). We further added a ball-like region $S_{V}$ (the RNA initial concentration will be located there), which is located within the cytosol, and a belt-shaped volumetric ribosomal region $R_{V}$ surrounding the ER surface, as shown in Figure 5. Hence, we have the volumetric domain

(1) $$\Omega =C_{V}\cup W_{V}\cup R_{V}\cup S_{V}$$

The part of the volume grid discretizing the ER lumen is removed because the ER lumen is not involved in the processes investigated in this work, i.e., because it does not contribute to the vRNA replication cycle.

Note that in opposite to the surface domains, the ribosomal region is not located at some sort of intersection of webs and ER, but moreover on a separated subdomain. This accounts for some uncertainties about the general question of the location of the ribosomes.

In the following, the volume grid will be used as basis for the development of partial differential equation (PDE) models of the vRNA replication cycle which take place within the volume of the computational domain rather then just the surfaces in contrast to the sPDE models we present here as well.

### 2.4. PDE Models

#### 2.4.1. The Surface PDE Model

The first model we develop is introduced on the aforementioned small cutout of the ER (see Figure 3c and Figure 4). This geometric setup is the basis for all surface models which we present in this paper.

The core of the new model is the fact that the NSPs anchor to the ER surface directly after their cleavage. Therefore, we have to restrict the movement of the NSPs to the ER surface. Further, since it is very difficult to capture the movement of the vRNA experimentally, and since it is not clear how the vRNA moves in reality, we make the assumption that the movement of the vRNA is restricted to the ER surface in the same way as the movement of the NSPs (the latter one was proven experimentally [1]).

Therefore, we create a model in which the movement of the NSPs *and* the movement of the vRNA are restricted to the ER surface.

Since the volume-to-surface ratio of the web regions is negligibly small, we assume that the diffusion process of the viral proteins can be reliably modeled to take place only on the ER surface and on the surface of the web regions. This is, however, a first approximation, since the viral proteins may in fact move both on the ER surface and *within* the web regions.

The mathematical description of the vRNA movement, polyprotein translation and cleavage, NSP movement and accumulation / binding at web protein regions, vRNA copying, and the movement of the new vRNA towards the ribosomal regions to produce new NSP are formulated by introducing the following concentrations:

- concentration of vRNA: R(x,t);
- concentration of the viral polyprotein: P(x,t);
- concentration of the Web Protein (i.e., the NSP which arises from the cleaved polyprotein and which accumulates to form the web regions) at the predefined webs: W(x,t);
- host factor concentration: H(x,t).

Indeed, the concept of these concentrations follows similar ideas of previous ODE models, e.g., [6,7,17,18]. However, by now, these concentrations are all regarded as functions of space ($x\in D\subset R^{3}$) *and* time. The spatio-temporal evaluation of *R*, *P*, *W*, and *H* is thus modeled by the nonlinear coupled surface PDEs (sPDEs)

(2a) $$\begin{array}{cc} \partial_{t}R & ={\mathrm{div}}_{T}\left(D_{R}\nabla_{T}R\right)+r_{1}WHR, \end{array}$$

(2b) $$\begin{array}{cc} \;\partial_{t}P & ={\mathrm{div}}_{T}\left(D_{P}\nabla_{T}P\right)+r_{2}R-r_{3}P, \end{array}$$

(2c) $$\begin{array}{cc} \;\partial_{t}W & ={\mathrm{div}}_{T}\left(D_{W}\nabla_{T}W\right)+r_{3}P, \end{array}$$

(2d) $$\begin{array}{cc} \partial_{t}H & ={\mathrm{div}}_{T}\left(D_{H}\nabla_{T}H\right)-r_{4}WHR, \end{array}$$

where $D_{R}$, $D_{P}$, $D_{W}$, and $D_{H}$ are the piecewise constant diffusion coefficients of the aforementioned quantities. We assume the diffusion coefficients to be piecewise constant in order to enable diffusion in those regions in which it is biologically supported that a substance moves and in order to forbid diffusion in those regions in which no movement of substance is allowed. For example, the polyproteins are not allowed to diffuse on the ER surface away from the ribosomes, the web proteins are not allowed to diffuse away from the web regions, neither on the ER surface nor into the cytosol.

We use the following notations: The tangential divergency is denoted as ${\mathrm{div}}_{T}$ and $\nabla_{T}$ is the tangential gradient operator [49]. The diffusion-reaction laws Equations (2a)–(2d) are surface partial differential equations (sPDEs). They describe the diffusion and reaction processes occurring on the membrane of the ER and the web surfaces. The reaction rates, $r_{1},r_{2},r_{3}$ and $r_{4}$, are piecewise constant functions, i.e.,

(3) $$\begin{array}{ccc} r_{1}\left(x\right)>0,\;\;\forall \;x\in W, & \; & r_{1}\left(x\right)=0,\;\mathrm{otherwise},\\ r_{2}\left(x\right)>0,\;\;\forall \;x\in R, & \; & r_{2}\left(x\right)=0,\;\mathrm{otherwise},\\ r_{3}\left(x\right)>0,\;\;\forall \;x\in R, & \; & r_{3}\left(x\right)=0,\;\mathrm{otherwise},\\ r_{4}\left(x\right)>0,\;\;\forall \;x\in W, & \; & r_{4}\left(x\right)=0,\;\mathrm{otherwise}, \end{array}$$

The biological meaning of the reaction constants $r_{1},r_{2},r_{3},r_{4}$ is the following:

- $r_{1}$ describes the rate of the polymerization of new vRNA in terms of the multilinear concentrations of vRNA, web protein and host factor;
- $r_{2}$ describes the rate of the polyprotein translation in terms of the linear concentration of vRNA;
- $r_{3}$ describes the rate of the cleavage of the polyprotein into web (accumulating) protein;
- $r_{4}$ describes the rate of host factor depletion during vRNA polymerization in terms of the multilinear concentrations of vRNA, web protein and host factor. Note that the vRNA polymerization and the host factor depletion are proportional with the only difference of the ratio of $r_{1}$ to $r_{4}$ to enable a different amount of host factor depletion compared to vRNA polymerization while vRNA gets copied.

The host factor *H* is introduced to avoid an unrealistic production of vRNA, which would be divergent otherwise (cf. also, e.g., [18]). The host factor is consumed in parallel when the vRNA gets replicated within the webs. Moreover, since the replication of a virus depends on the possibility to hijack the metabolism of the host (in fact, no replication may occur if no host factor is available), the introduction of a host factor is necessary to make the model realistic.

To model the fact that the polyprotein does not diffuse outside R, whereas the web protein does not diffuse outside W∪R, we choose $D_{P}$ and $D_{W}$ as

(4a) $$\begin{array}{cccc} D_{P}\left(x\right)>0, & \;\;\forall \;x\in R, & \;\; & D_{P}\left(x\right)=0,\;\mathrm{otherwise}, \end{array}$$

(4b) $$\begin{array}{cccc} D_{W}\left(x\right)>0, & \;\;\forall \;x\in W\cup R, & \; & D_{W}\left(x\right)=0,\;\mathrm{otherwise}. \end{array}$$

Furthermore, we prescribe the initial conditions

(5a) $$\begin{array}{cc} R(x,0) & =\left\{\begin{array}{cc} \;R_{0} & \mathrm{in}R_{i_{⋆}},\mathrm{with}i_{⋆}\in \left\{1,\ldots ,7\right\}\;\left(\mathrm{we}\;\mathrm{choose}\;i_{⋆}=2\right),\\ \;0 & \mathrm{in}D\backslash R_{i_{⋆}}, \end{array}\right. \end{array}$$

(5b) $$\begin{array}{cc} W(x,0) & =0\;\mathrm{in}D, \end{array}$$

(5c) $$\begin{array}{cc} H(x,0) & =1\;\mathrm{in}D, \end{array}$$

(5d) $$\begin{array}{cc} P(x,0) & =0\;\mathrm{in}D. \end{array}$$

All surface PDEs of this paper are solved by employing Neumann zero boundary conditions on ∂D for each one of the concentrations.

We emphasize that, although fitting of experimental data is necessary to obtain physically meaningful values of $D_{R}$, $D_{P}$, $D_{W}$, $D_{H}$, $r_{1}$, $r_{2},r_{3}$, and $r_{4}$, we could prescribe here only “heuristic” values for these quantities, because we had no access to suitable experimental data. We also assume that in part, adequate data are not available so far and ask for new experiments.

Since the parameters $D_{P}$, $r_{3}$, and $r_{2}$ are such that the polyprotein decays (i.e., cleaves) very quickly in comparison with the other species, the asymptotic behavior of the system is given by

(6a) $$\begin{array}{cc} \partial_{t}R & ={\mathrm{div}}_{T}\left(D_{R}\nabla_{T}R\right)+r_{1}RWH \end{array}$$

(6b) $$\begin{array}{cc} \partial_{t}W & ={\mathrm{div}}_{T}\left(D_{W}\nabla_{T}W\right)+r_{2}R \end{array}$$

(6c) $$\begin{array}{cc} \partial_{t}H & ={\mathrm{div}}_{T}\left(D_{H}\nabla_{T}H\right)-r_{4}RWH \end{array}$$

Note that the reaction term $r_{1}RWH$ is a multilinear function of R,W and *H*, as this is the case for other ODE-based models (cf., e.g., [5,6,17]). Still, this approximation is possible as long as the concentrations R,W and *H* are sufficiently small.

Given a (scalar) physical quantity f:D→R and a non-empty set Υ∈D, we write ${\left[f\right]}_{\Upsilon }$ to indicate the restriction of *f* to the set in which it is nonzero. This is done to visualize more clearly when *f* has to be evaluated. Hence Equations (6a)–(6c) become

(7a) $$\begin{array}{cc} \partial_{t}R & ={\mathrm{div}}_{T}\left(D_{R}\nabla_{T}R\right)+{\left[r_{1}RWH\right]}_{W} \end{array}$$

(7b) $$\begin{array}{cc} \partial_{t}W & ={\left[{\mathrm{div}}_{T}\left(D_{W}\nabla_{T}W\right)\right]}_{W\cup R}+{\left[r_{2}R\right]}_{R} \end{array}$$

(7c) $$\begin{array}{cc} \partial_{t}H & ={\mathrm{div}}_{T}\left(D_{H}\nabla_{T}H\right)-{\left[r_{4}RWH\right]}_{W} \end{array}$$

Further, since we choose piecewise constant diffusion coefficients, the surface diffusion is governed by the Laplace-Beltrami operator $\Delta_{T}$, i.e., the projection of the Laplace operator to the tangential space of the two dimensional ER-hypersurface E, which is embedded into the complete 3D space [49]. Hence, the equations may be rewritten in the simple form

(8a) $$\begin{array}{cc} \partial_{t}R & =D_{R}\Delta_{T}R+{\left[r_{1}RWH\right]}_{W} \end{array}$$

(8b) $$\begin{array}{cc} \partial_{t}W & ={\left[D_{W}\Delta_{T}W\right]}_{W\cup R}+{\left[r_{2}R\right]}_{R} \end{array}$$

(8c) $$\begin{array}{cc} \partial_{t}H & =D_{H}\Delta_{T}H-{\left[r_{4}RWH\right]}_{W} \end{array}$$

The original Equations (2a)–(2d) incorporating the intermediate polyprotein state may be rewritten in this way as

(9a) $$\begin{array}{cc} \partial_{t}R & =D_{R}\Delta_{T}R+{\left[r_{1}RWH\right]}_{W}, \end{array}$$

(9b) $$\begin{array}{cc} \partial_{t}P & ={\left[D_{P}\Delta_{T}P+r_{2}R-r_{3}P\right]}_{R}, \end{array}$$

(9c) $$\begin{array}{cc} \partial_{t}W & ={\left[D_{W}\Delta_{T}W\right]}_{W\cup R}+{\left[r_{3}P\right]}_{R}, \end{array}$$

(9d) $$\begin{array}{cc} \partial_{t}H & =D_{H}\Delta_{T}H-{\left[r_{4}RWH\right]}_{W}. \end{array}$$

##### Degrees of Freedom (DoFs):

The node number and the number of the degrees of freedom (DoFs) of the surface PDE evaluations at base level is shown in Table 1. The simulations presented afterwards are performed within a one-fold spatial refinement multigrid environment. The DoF number is about 6000 times the number of concentrations. For example, the simple model with 4 concentrations Equations (2a)–(2d) has about 20,000 DoFs at base level and about 90,000 DoFs at one fold refinement (see Table 1).

##### Parameter Set and Variations

The standard parameter set of the sPDE model as computed by means of Equations (8a)–(8c) respectively Equations (9a)–(9d) is depicted in Table 2.

To test for the sensitivity of the model to the parameter variation, we will change various of the values in Section 3.

We change only one variable within our examples as evaluated within Section 3, while the others keep their standard values. Anyhow our model is not restricted to these values. In particular, our program code accepts any value for the computations. The values chosen in this study are just for demonstrating the potential of the model and the sensitivity to parameter changes.

The number of webs arises from the microscopy data and we may only “switch off” some of them, but we do not add artificially.

#### 2.4.2. The “Volume” PDE Model

In opposite to the surface PDE model as introduced before, we introduce a model where the movement of all concentrations takes place within the cytosol volume and the web volumes rather then the ER and web surfaces.

On top of the volume meshed geometry as described in Section 2.3, we study the diffusion and reaction processes of four substances within the volume mesh Ω (as depicted in Figure 5): viral RNA (“RNA”—*R*), nonstructural protein (“NSP”—*N*), web bound viral protein (“web”—*W*) and host factor (“host”—*H*):

(10a) $$\begin{array}{cc} \partial_{t}R & =D_{R}\Delta R+r_{1}RWH \end{array}$$

(10b) $$\begin{array}{cc} \partial_{t}N & =D_{N}\Delta N+r_{2}R-r_{3}N \end{array}$$

(10c) $$\begin{array}{cc} \partial_{t}W & =D_{W}\Delta W+r_{3}N \end{array}$$

(10d) $$\begin{array}{cc} \partial_{t}H & =D_{H}\Delta H-r_{4}RWH \end{array}$$

where

(11) $$\begin{array}{cccc} r_{1}\left(x\right)>0, & \;\;\forall \;x\in W_{V}, & \; & r_{1}\left(x\right)=0,\;\mathrm{otherwise},\\ r_{2}\left(x\right)>0, & \;\;\forall \;x\in R_{V}, & \; & r_{2}\left(x\right)=0,\;\mathrm{otherwise},\\ r_{3}\left(x\right)>0, & \;\;\forall \;x\in W_{V}, & \; & r_{3}\left(x\right)=0,\;\mathrm{otherwise},\\ r_{4}\left(x\right)>0, & \;\;\forall \;x\in W_{V}, & \; & r_{4}\left(x\right)=0,\;\mathrm{otherwise},\\ D_{W}\left(x\right)>0, & \;\;\forall \;x\in W_{V}, & \; & D_{W}\left(x\right)=0,\;\mathrm{otherwise}, \end{array}$$

where $W_{V}$ is the (tetrahedralized) volume subdomain inside the boundaries of the reconstructed web regions and $R_{V}$ the tetrahedralized volume subdomain of the ribosomal belt. $D_{R},D_{W},D_{N},D_{H}$ are the respective diffusion constants.

The biological meaning of the reaction constants $r_{1},r_{2},r_{3},r_{4}$ is the following:

- $r_{1}$ describes the rate of the polymerization of new vRNA in terms of the multilinear concentrations of vRNA, web protein and host factor;
- $r_{2}$ describes the rate of the translation of NSPs in terms of the linear concentration of vRNA;
- $r_{3}$ describes the binding rate the NSPs to the web regions forming web (accumulating) protein;
- $r_{4}$ describes the rate of host factor depletion during vRNA polymerization in terms of the multilinear concentrations of vRNA, web protein and host factor. Note that as in the case of the sPDE model before, the vRNA polymerization and the host factor depletion are proportional with the only difference of the ratio of $r_{1}$ to $r_{4}$ to enable a different amount of host factor depletion compared to vRNA polymerization while vRNA gets copied.

We use initial and boundary conditions

(12a) $$\begin{array}{cc} R(x,t=0) & =\left\{\begin{array}{cc} \;R_{0} & \forall x\in S_{V}\\ \;0 & \mathrm{else}\;\mathrm{in}\;\Omega \end{array}\right.\\ N(x,t=0) & =0\;\forall \;\forall x\in \Omega \end{array}$$

(12b) $$\begin{array}{cc} W(x,t=0) & =0\;\forall \;\vec{x}\in \Omega \end{array}$$

(12c) $$\begin{array}{cc} H(x,t=0) & =H_{0}\;\forall \;\vec{x}\in \Omega \end{array}$$

(12d) $$\begin{array}{cc} H(x,t) & =H_{0}\;\forall \;x\;\in \partial \Omega \wedge \forall t \end{array}$$

where $S_{V}$ is the small sphere inside the cytosol which acts as original source of the vRNA. This is in opposite to the surface models where the RNA initial condition was already located at the ribosomes. Thus, this approach takes into account one single step more at the beginning, which however is not so important at this stage. However, this step reminds us of a major question of RNA movement modeling—“how does the viral RNA move?”—This seems to be not completely clarified experimentally to our understanding of the experimental literature.

All other boundary conditions are Neumann zero and all concentrations (besides the vRNA) are zero at the beginning. The Neumann zero condition does not apply to the host factor for which we use constant Dirichlet values at the outer boundary of the computational domain.

Summarizing the afore derived formlae, the RNA start concentration is located at a small ball-like region not far away from the ribosome belt around the ER. At the ribosomic belt, the RNA may translate NSPs. To this end, the dynamics of the viral components takes place within the volumetric region of cytosol and the web regions and reads in the (volume) PDE description. (Sometimes we will call the PDEs in the complete space “volume” PDEs (vPDEs) to distinguish from the afore mentioned surface PDE model.) We mention that in opposite to the surface models from before, we have now a freely diffusing NSP “*N*” which gets bound at the web regions. Once bound at the web regions, the web protein (or web accumulating protein) is denoted as “*W*”. Finally, the web protein replicates the vRNA which again moves to the ribosomes to translate the NSP. The vRNA cycle is closed and continues.

We use the afore introduced notation to make the equation structure more obvious. For a given quantity f:D⟶R and a set Ξ∈Ω we write ${\left[f\right]|}_{\Xi }$ to indicate that *f* is nonzero only in Ξ. In this way, it is easy to visualize when *f* has to be evaluated. Using this definition of the pipe applied to the corresponding terms, the equations read

(13a) $$\begin{array}{cc} \partial_{t}R & =D_{R}\Delta R+\left[r_{1}RWH\right]{|}_{W_{V}} \end{array}$$

(13b) $$\begin{array}{cc} \partial_{t}N & =D_{N}\Delta N+\left[r_{2}R\right]{{|}_{R_{V}}-\left[r_{3}N\right]|}_{W_{V}} \end{array}$$

(13c) $$\begin{array}{cc} \partial_{t}W & ={\left.\left[D_{W}\Delta W+r_{3}N\right]\right|}_{W_{V}} \end{array}$$

(13d) $$\begin{array}{cc} \partial_{t}H & =D_{H}\Delta H-\left[r_{4}RWH\right]{|}_{W_{V}} \end{array}$$

We mention that the Laplace operator in the 3D volume has a very simple representation:

(14) $$\Delta =\frac{\partial^{2}}{\partial x^{2}}+\frac{\partial^{2}}{\partial y^{2}}+\frac{\partial^{2}}{\partial z^{2}}$$

We perform the computations with one-fold spatial refinement of the basic unstructured grid. The overall number of mesh elements, nodes, and degrees of freedom (DoFs) are listed in Table 3.

The parameter set used for the volume PDE evaluations is reported in Table 4.

### 2.5. Comparison to State-of-the-Art ODEs

All presented models of diffusion-reaction sPDEs and PDEs can be considered to be some sort of “prolongation” of the standard-ODE models of HCV vRNA dynamics (such as presented, e.g., in [5,6,7,8,9,10,11]) to the 3D space. For the sake of simplicity however, we focus so far on less ingredients in comparison to the standard approaches.

### 2.6. Technical Evaluation

Our volume PDEs are discretized by means of standard vertex-centered finite volume (FV) methods [50].

Surface PDE discretization within the Finite Volume/Finite Element (FV/FE) environment is performed in principle as in the “standard” PDE case: We discretize in a manner similar to [51,52]. In contrast to these approaches we use the vertex-centered finite volume method, cf. [40]. The computational domain is repartitioned by a dual grid consisting of a small non-overlapping set of control volumes (Although the control "volume" is actually a surface for the sPDEs, we still call it “volume” to keep consistent with the standard notation of FV). Each control volume contains exactly one vertex of the grid. The balance law is ensured by the numerical scheme and the fluxes from and into the control volumes are balanced. For the usage on 3D embedded 2D surfaces, the control volumes are now constructed with respect to the manifold. The control volumes are lower-dimensional parts of the manifold and normals on the boundary of the control volumes are chosen to be tangential to the surface.

The resulting nonlinear equation system is solved by means of a nonlinear Newton Solver. We apply a BiCGStab solver, preconditioned with a Geometric Multigrid Solver (GMG), as linear solver of the Newton solver.

The simulations of the afore derived sPDE models and of the (volume) PDE model (with ER lumen exclusion) were performed with the UG4 framework [34,36]. This allows us to evaluate the concentrations of each ingredient at any temporal and especially also at any spatial point of the computational domain, i.e., of the cutout part of the hepatocyte.

### 2.7. Remarks upon the Numerical Stability

Since the present models are qualitative rather then quantitative, there was no need for extended studies of grid convergency of the results. Within our qualitative approach, the computations are performed for one-fold spatial refinement of the unstructured surface and volume grids and variable time step size. However, for upcoming quantitative computations, intensive grid refinement checks will get necessary to test for the refinement stability, i.e., when experimental data are available for comparisons [40].

## 3. Results

In the following, we present the results related to the mathematical models discussed in the previous sections. In particular, we focus on the formulations based on the set of Equations (8a)–(8c), (9a)–(9d) and (13a)–(13d). We emphasize that all the considered models are computed on realistic geometries, reconstructed on the basis of the microscopy data presented in Section 2.2.

In the following, we will sometimes denote the viral RNA simply as RNA rather then vRNA. Since we do not refer to body-own messenger RNA at any point, this should not cause confusion.

### 3.1. Surface PDE Model of vRNA, webProtein and Host Factor

This model is based on Equations (8a)–(8c).

**Figure 6:**
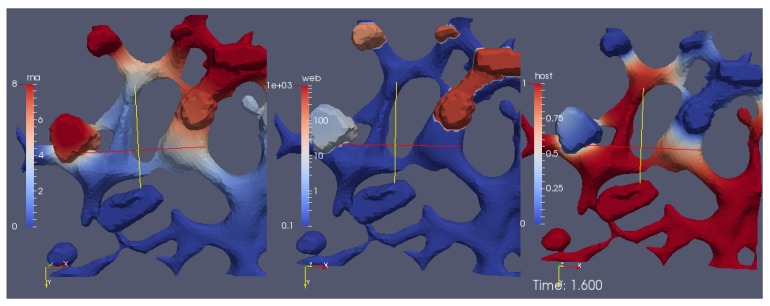
Screenshot of the movie S1 Video in Supplementary Material: Simulation of vRNA, NSP, web protein and host factor interplay dynamics on cutting plane version of rotating ER surface. Simulation of model Equations (8a)–(8c), described in detail in Section 3.1.

A screenshot of the movie S1 Video in Supplementary Material reporting the evolution of the surface concentrations of the vRNA, web-protein, and host factor (denoted in the formla by *R*, *W*, and *H*, respectively) is shown in Figure 6. We emphasize that *R*, *W*, and *H* are the solutions of Equations (8a)–(8c), respectively, obtained with the initial conditions Equations (5a)–(5c).

The movie depicts a portion of the computational domain obtained by cutting the domain itself by means of plane. This is done to permit the visualization of the initial concentration R(x,0), which otherwise cannot be seen, since it is nonzero only at the ribosomes (i.e., at the intersections between the ER and the web regions, which are indeed hidden unless the computational domain is disclosed by a cutting plane).

With reference to Figure 6, the concentrations are ordered in the following way: From left to right, one can see a snapshot of the spatial distribution of the concentration of the vRNA, *R*, web protein, *W*, and host factor, *H*. (This means that the left sector of the screen represents the RNA, the middle sector the web protein, and the right sector of the movie the host factor concentration.) As soon as we start the simulations, it is possible to observe that the web protein is produced at the ribosomes (cf., middle sector). Indeed, the RNA start concentration (visible at the left sector of the simulation movie screen) causes the translation of the web protein (visible in the middle sector of the simulation movie screen). Then, the web protein moves into the web region (middle sector), which—as elsewhere recalled—has been constructed on the basis of experimental data [29].

Since at the beginning of the simulation we watch the section of the ER from behind, we are not able to see the evolution of the concentrations inside the web regions that find themselves over the ER surface. To visualize such evolution, we rotate the perspective to see the ER from the front.

While the perspective is still rotating, we can already see that the web protein (which gets translated by to the RNA at the ribosomes) moves into the web region (cf., middle sector). We can observe this process because the color of the web protein concentration changes from blue (low concentration) to red, which indicates high concentration. However, we observe also that the RNA itself is diffusing. The RNA diffuses away into two directions: One direction of the RNA diffusion happens on the ER surface. However, the concentration gets rather small there soon. Anyhow, the other direction of RNA diffusion is into the web region. Inside the web region, the major process takes place: The RNA concentration rises up again, because the web proteins copy the RNA. By now, the camera perspective does not move any more. From now on until to the end of this simulation movie, the perspective of the view is such that we observe the processes so that we see the front of the ER and the web regions. One sees clearly in the left sector of the movie, how the RNA concentration is uprising strong within the web region. The web is already growing, cf., middle sector of the movie. At the right sector of the movie, we see the host factor which, due to the RNA production, gets consumed. By now, the web region is filled with RNA. This is visible due to the red color of the RNA sector of the three concentrations, i.e., in the left sector of the movie. Due to the diffusion, now the RNA moves strongly outside again to the ER surface. This movement is visible on the ER surface because the red color appears on the ER surface of the RNA representing sector of the scene. Once the diffusing RNA arrives at the next experimentally predefined web region, the RNA also arrives at the corresponding ribosomes which are located at the intersection of the reconstructed ER surface and the reconstructed web region. Therefore, the ribosomes are "hidden" and not visible any more from our present perspective. Now, the same game begins again as described before: The RNA produces the web proteins. In the middle sector of the clip, i.e., the web protein concentration sector, one sees that the web is growing, indicated by the change of the color from blue to red within this (second) web region. The RNA diffuses (as before at the other web) into the new web region. There, the web protein replicates the RNA as before at the other web. And as before, one observes the depletion of the host factor (right sector). The newly synthesized RNA diffuses away again and spreads itself further. The RNA reaches the next web region, translates the web protein which replicates the RNA, and so on—in this way, some sort of “wave”’propagates within the cell which causes the RNA replication, the growth of the webs, and the host factor depletion. As soon as the host factor is depleted, the RNA reproduction stops at the corresponding sites. This stop appears especially once when also the host factor of the surrounding regions is depleted and no further host factor can diffuse to the replication sites, due to the lack of the availability. In this manner, the “wave” contaminates the cell bit by bit with viral RNA and viral web regions. At the beginning, one sees clearly that the viral RNA is concentrated at the web regions. Only after when a lot of RNA is produced, after a substantial amount of time, the complete computational domain (i.e., the cutout of the cell where we evaluate the model) at the left hand side sector (the RNA part) appears to be red, i.e., filled with RNA. Indeed, in particular at this stage, the model still bears the potential to be improved. This improvement could be performed by means of the incorporation of a degradation of RNA, which should take place especially exteriorly of the web regions, i.e., at the ER surface.

As mentioned before, the simulations assume that a host factor is consumed during the reproduction of the viral RNA. Of course, this host factor will be missed at another place, namely for the normal metabolic processes of the cell. Indeed, the cell does not produce the host factor for the sake of virus reproduction but moreover for metabolic purposes. However, due to the depletion of the host factor and presumably also due to the overall appearing replication sites of the vRNA (the web regions), the cell will get into cell stress. The depletion of the host factor, the web regions and the appearance of the vRNA for sure are not beneficial for the cell and its metabolism.

To this end, our simulations recapitulate already by now the major events of the vRNA cycle as observed experimentally. Since the vRNA reproduction of our model is also proportional to the host factor, a breakdown of vRNA or NSP production cannot take place which else would appear and which would not be physically sound.

#### 3.1.1. Quantitative Spatially Resolved Evaluation

**Figure 7:**
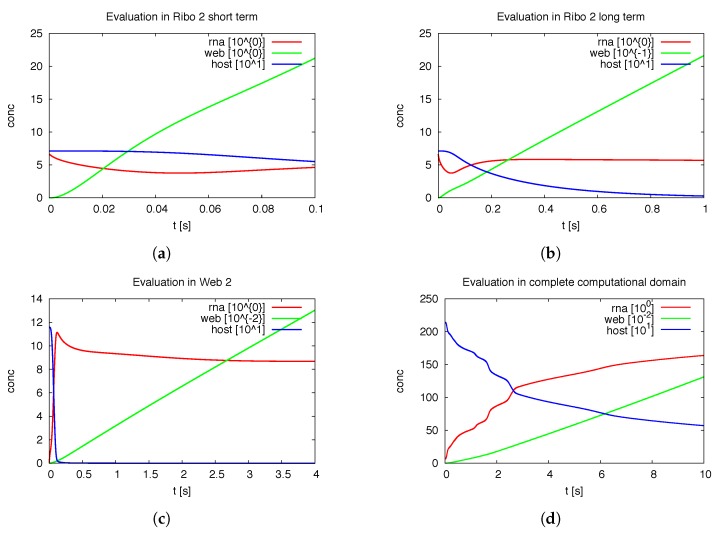
Spatially resolved sPDE model evaluation (Equations (8a)–(8c)) of vRNA (rna), Web Protein (web) and host factor (host) concentrations separated by subdomains (**a**–**c**) and complete computational domain (**d**), examples using heuristic values for diffusion constants and initial concentrations. Note: The complete computational domain refers as the cutout of the cell rather then the complete cell.

Figure 7 shows the deployment of the integrals of the concentrations within special subdomains, also resolved within different time scales. We show the development of the three concentrations of vRNA, web protein and host factor for the short time behavior at the beginning of the process within the first ribosomal region, where the vRNA is located at the very beginning. Further we depict the long time behavior at the same location. As well, we depict the deployment of the concentrations within the attached web region. Finally, we show the deployment of the integrals of the concentrations evaluated within the *complete* domain of computation. In future work, our modeling framework has to be extended to complete cells and the model has to be further improved, namely also adapted to experimental values. Then, the integration of the concentrations over the complete cell will get an important benchmark test by means of the comparison to already by known known data averaged over the complete cell. For example, [39] reported data concerning the deployment of the numbers of different viral proteins and the vRNA for single cells.

Of special interest is the observation of the step structure in the RNA dynamics as visible in Figure 7d: Each time that the RNA reaches a new web, the overall RNA level develops some sort of step-wise increase. The “step-structure” of the RNA increase is due to the successive involvement of new web regions. Hence, the influence of the spatial distribution of the membranous web growth manifests itself trough the “staircase”-shape of the curves of the integrated concentrations of RNA and the host factor, as visible in Figure 7d. It would bring new insight to observe such effects within fluorescence data of single cells. Anyhow such an observation will not be possible within Western/Northern-blot analysis based homogenized experiments. Spatially homogenized descriptions did not predict such structures up to now to our best knowledge.

It would be also interesting to investigate experimentally weather the propagation of the RNA through the cell causes some sort of ordered “switching on” of new webs, or if the RNA induced translation and web growth appears randomly all over different places of the cell. Fluorescence experiments presumably have the potential to investigate this property. Such research would help to justify our simple RNA diffusion approach or would ask for more involved RNA dynamics descriptions, thereby helping to resolve the so far experimentally unresolved question of the RNA movement properties.

#### 3.1.2. Variation of the Parameters and Web Numbers

**Figure 8:**
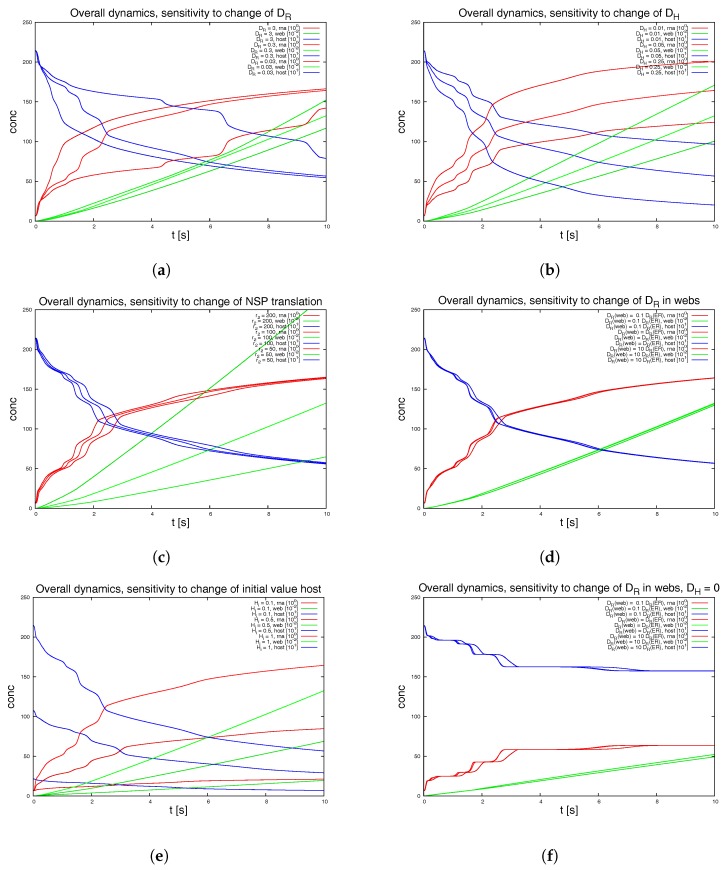
Variation of parameters in Equations (8a)–(8c). Note: Always only one parameter gets varied, all others are kept fixed and take the values as reported in Table 2, besides in case f. Variations: (**a**) Diffusion constant of the RNA $D_{R}$; (**b**) Diffusion constant of the host $D_{H}$; (**c**) NSP (i.e., web protein) translation reaction rate $r_{2}$; (**d**) Diffusion constant of the RNA inside the webs $D_{R}{|}_{W}$; (**e**) initial value of Host, H(x,0) at all spatial points of the computational domain; (**f**) Switching off host factor diffusion $D_{H}=0$ and varying diffusion constant of RNA inside the webs $D_{R}{|}_{W}$. All parameters not denoted are as indicated in the standard parameter set, cf., Table 2.

**Figure 9:**
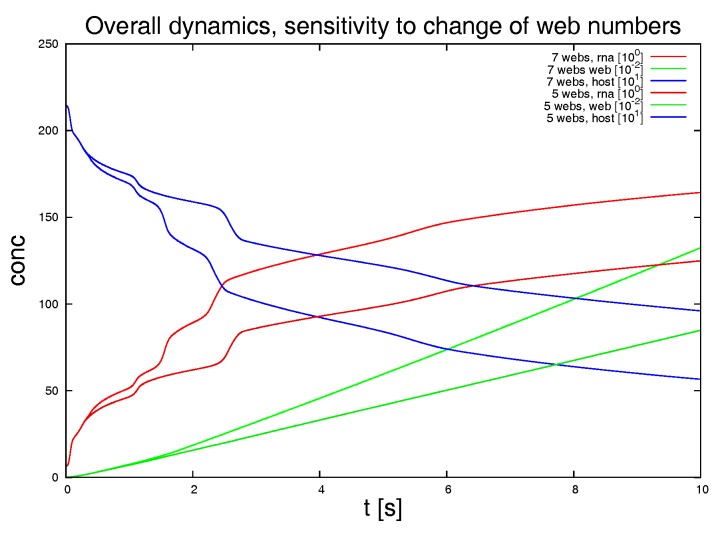
Switching off two of seven webs (web number 3 and 5) to test for the influence of the web density. Indicates effective change of the experimental data as base of the geometric setup. This case is shown separately since it is only based in part upon the geometry as derived from experiment. All other parameters from standard parameter set, cf. Table 2.

To test for the sensitivity of the model, we vary the parameters featuring in the sPDEs. Figure 8 depicts various examples. We also switch off some of the webs in order to mimic a less dense web. Figure 9 shows the result of this study. The latter approach is non-biological and just for test purposes, while the other variations allow for insight into the structure of the sPDE model upon the realistic reconstructed geometric structures.

The graphical representations show the integrals of the concentration of RNA, web and host factor for the complete computational domain. Hence they repeat those results of Figure 7d which depicted the overall dynamics. These overall kinetics behaviour also gives the base for comparisons to ODE models.

We remark the following major observations:

- Figure 8a: For the initial dynamical phase, the variation of the diffusion coefficient of the RNA has a major influence upon the RNA dynamics and the host factor depletion, while it has no major influence upon the long term level. The web growth however is only affected in a minor manner by the RNA diffusion coefficient.
- Figure 8b: The diffusion constant of the host factor has a very important influence upon the complete kinetics.
- Figure 8c: The reaction of the translation of NSPs (i.e., web protein, since intermediate polyprotein not considered) has an influence at the short term level each time that the RNA reaches a new web, but even in the middle-term level, this influence is nearly negligible.
- Figure 8d: The influence of a pure change of the diffusion coefficient of the RNA inside the webs (keeping the diffusion on the ER as before) has nearly no influence upon the kinetics as long as the host factor may diffuse with a substantial diffusion coefficient.
- Figure 8e: The initial value of the host factor has strong influence on the overall dynamics, but this influence may be considered to be even less important compared to the afore described influence of the transport of the host factor.
- Figure 8f: Once the host factor diffusion is switched off, it is interesting to note that at a short-term level, the change of the RNA diffusion coefficient inside the webs shows substantial impact at the short term level, but not at the middle term level.

The latter observation indicates that the afore described, nearly vanishing influence of the variation of the RNA diffusion coefficient inside the webs arises from the superposed influence of the diffusion of the host factor. This argument also explains why the NSP translation rate has only minor influence upon the overall dynamics as demonstrated in Figure 8c).

Switching off 2 of the 7 web regions, as shown in Figure 9, demonstrates the strong influence of the density of web regions (i.e., number of web regions divided by the area of the considered volume) on the dynamics.

### 3.2. Surface PDE Model Incorporating the Polyprotein

In the last section, we have presented the deployment of the model with 3 concentrations (vRNA, web protein and host factor). On this way, we have neglected that the web protein does not get translated directly. Indeed, at first the polyprotein gets translated Equation (2b) and cleaves into the NSPs, namely the web protein Equation (2c).

Therefore, we consider this process again, in a complete manner including also the intermediate step of polyprotein translation and cleavage as indicated by the original full model of Equations (2a)–(2d) (respectively Equations (9a)–(9d), rewritten in a more intuitive form). This means that we describe the evaluation of the surface model of vRNA, polyprotein, web (accumulating/accumulated) protein and host factor.

**Figure 10:**
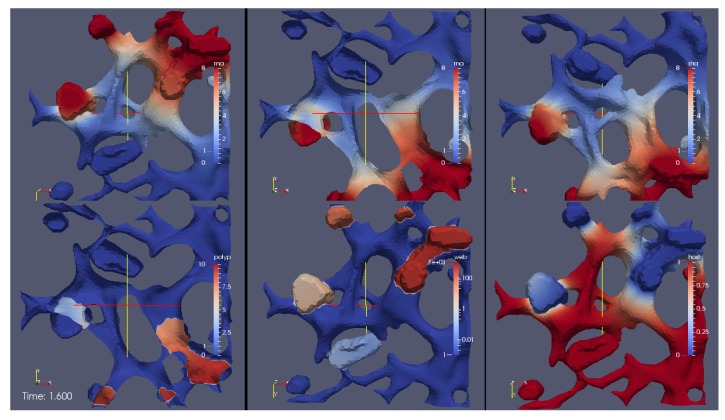
Screenshot of the attached movie S2 Video in Supplementary Material of the simple surface model including the intermediate polyprotein state. Simulation of model Equations (9a)–(9d), description cf. Section 3.2.

The corresponding simulation movie S2 Video in Supplementary Material shows the deployment of the concentrations of vRNA, polyprotein, web protein and host factor. Figure 10 depicts a screenshot of this movie. Watching this movie, the dynamics is overall similar as in Section 3.1. In contrast to before, this time the perspective is static rather then rotating. Therefore, a part of the concentrations is shown from different perspectives, i.e., some concentrations occupy more then one sector of the movie, hence are shown simultaneously from different (static) perspectives:

All three upper sectors show the deployment of the RNA, but from different perspectives. The lower three sectors depict (from left to right) the polyprotein, the web protein and the host factor.

Let us explain in detail the different perspectives of the movie sectors:

At top in the middle (one of three RNA sectors), one sees the geometry from the same perspective as shown at the beginning of the last Section 3.1, i.e., as in movie S2 Video in Supplementary Material: The perspective is from behind to the inner of the ER because we use again a cut plane for “opening” the ER (in the perspective, not in the simulation—the simulations are performed on the complete computational domain in all cases). This means that we may see the RNA initial concentration in the same way as we have seen it at the beginning of movie S2 Video in Supplementary Material.

At the upper sector on the left, we observe the RNA at the same perspective which we have seen in movie S2 Video in Supplementary Material *after* the rotation, i.e., we look from the front to the ER and the web regions.

At the sector to the upper right, we observe the RNA concentration from behind, but without the cut plane, i.e., from the same perspective as in the middle, but *without* the cut plane.

Therefore, we can observe the RNA deployment from nearly all possible directions including cut planes. The only “missing” direction would be from the front with a cut plane, but we omit this for the sake of simplicity.

At the lower left sector, we watch the polyprotein from behind with a cut plane (as the RNA upper in the middle). In the lower middle sector, we observe the web (accumulating) protein from the front view (as the upper left RNA), and on the right sector, we observe the host factor from the same front perspective.

Having described the preconditions of the simulation movie, we want to evaluate the simulation itself:

At the beginning, we see that the RNA (visible in the upper middle sector) translates the polyprotein (visible in the lower left sector) at the ribosomal region. The polyprotein cleaves immidiately into the web (accumulating) protein, causing the growth of the web (visible at the lower middle sector).

This causes the reproduction of the RNA within the web regions (visible preferably upper left, but also upper middle sectors). The RNA diffuses away and reaches the next ribosomal region (visible at the upper left sector and upper middle sector). Hence, the polyprotein gets translated also at this ribosomal region into the web (accumulating) protein (visible in the lower middle sector), the web protein diffuses into the web region and causes the reproduction of the RNA (visible preferably upper left but also upper middle sectors).

Again, as before, the RNA-“wave” propagates through the geometry and the host factor gets consumed at those places where RNA gets polymerized (visible lower right sector).

In the sector at the upper right (RNA concentration), one observes only at later time the RNA production at the corresponding web regions. This refers to the condition that those web regions which are located such that they are visible from the perspective of the upper right are not reached by RNA at the very beginning. Therefore, from the perspective of this sector, at the beginning one observes only few free diffusing RNA but no RNA which arises from reactive production. However, this changes once the RNA reaches the web regions visible form this perspective (respectively the “hidden” ribosomes corresponding to the interface of web regions and ER surface), then also there the polyprotein gets translated, cleaves into web protein, and the web protein accumulates at the web regions, forming the webs which replicate the RNA and so on.

Strikingly, only little of the polyprotein is visible over the complete passage of time. This is due to the fast decay of the polyprotein into the web protein. This observation justifies the approximation as realized within the section afore Section 3.1, i.e., the model of only three concentrations.

Anyhow this approximation only makes sense as long as we consider only one non structural protein (NSP), which we denoted in this case as web protein (respectively sometimes web accumulating protein to be more precise). Once we extend the model thus that the polyprotein cleaves into various NSPs, we need the intermediate step of the polyprotein.

### 3.3. The Volume PDE Model

Like the models presented afore, the simulation of the mathematical model as described in Section 2.4.2, i.e., Equations (10a)–(10d), capture the major events of the vRNA reproduction of HCV in a fully spatio-temporal resolving manner. In this model, we permit the dynamics within the volume of cytosol and of the web regions with the exclusion of the ER lumen. Therefore, the focus lies on different aspects compared to the surface models. The major disadvantage of this volume model is that, so far, the anchoring of the NSPs to the ER surface is neglected and replaced by a full volume movement. The latter one could be more appropriate for the host factor characteristics and maybe also for the movement characteristics of the vRNA.

We calculate the PDEs for the interplay of RNA (“*R*”), NSP (“*N*”), web accumulating/bound protein “*W*” and a generic host factor “*H*” as regulator which gets consumed when the web protein reproduces the vRNA at the membranous webs. The model is evaluated in the domain which consists of cytosol, web and ribosomes, i.e., the afore explained 3D continuum (Section 2.3), i.e., the volume of the cytosol (with the ER lumen exclusion) and the web regions.

**Figure 11:**
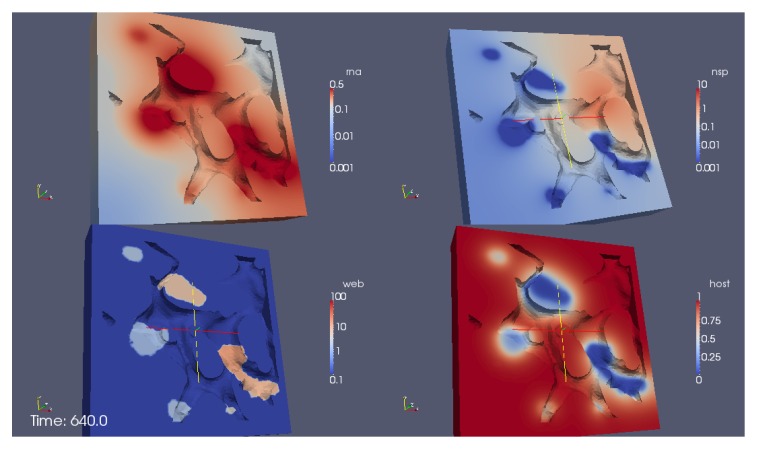
Screenshot of the attached supplemental movie S3 Video in Supplementary Material aiming the (volume) PDE model simulation (Equations (10a)–(10d) at the cell geometry where the ER lumen is excluded). For details, cf. Section 3.3. Brief description: The RNA diffuses away from the starting ball-like region, produces NSPs at the ribosome belt, the NSPs diffuse away and bind to the pre-defined web regions (as indicated by the reconstructions) as web protein. Inside the webs, the web (bound) protein “waits” for the RNA which diffuses there. The bound web protein replicates the vRNA at the membranous web region, the RNA diffuses back to the ribosome belt and the cycle is closed and continues.

Figure 11 shows a screenshot of the simulation movie S3 Video in Supplementary Material of the process using heuristic parameters. The complete movie visualizing the simulation from a cut perspective of the volume is attached as supplemental movie S3 Video in Supplementary Material. The perspective of the visualization is such that we consider the volume of computation disclosed by means of a cut plane. We visualize the RNA (upper sector at left), the NSP (upper sector right), the web protein (lower sector left) and the host factor (lower sector right).

In detail, we observe the following processes during the simulation:

The process starts directly after the uncoating of the first vRNA from the infecting virus. Therefore the simulations begin with an initial concentration of uncoated vRNA which is located originally within a sphere inside the cell close to ribosomes. The vRNA diffuses from its starting sphere and reaches the ribosomic region. (The ribosomic region has belt-like shape. It is not based on data. Moreover, we put the ribosomic belt “by hand” around a region of the ER). At the ribosomes, nonstructural viral proteins (NSPs) are translated. The NSPs diffuse away from the ribosomes and reach the experimentally predefined web regions. At these web regions, the NSPs get bound and form the membranous web complexes. The bound NSPs are denoted to be “web proteins” (web/webP). Since also the vRNA is diffusing within the cell, once vRNA reaches the membranous web complexes, vRNA gets reproduced. The new vRNA moves again to the ribosomes where the NSP translation mechanism gets enhanced. Also these NSPs diffuse away and reach the web regions. At the web regions, they get also bound as web protein. Since the web regions grow in this way, vRNA reproduction is further enhanced. A generic host factor gets consumed once vRNA gets polymerized and once the host factor is consumed, vRNA reproduction stops. (In this way, a breakdown of vRNA production is prevented, which also is biophysically sound—an unlimited production would not be realistic, and the energy which is needed to polymerize new vRNA for sure has to be strongly related to the host environment.)

**Figure 12:**
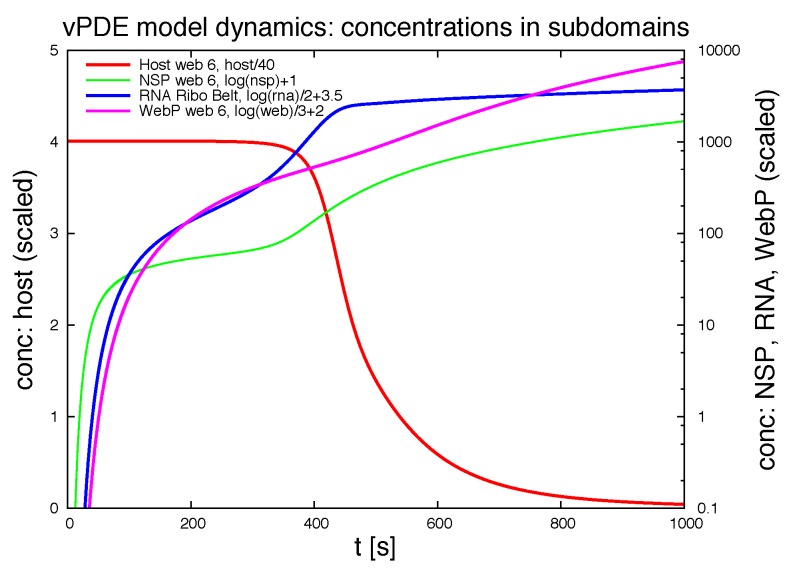
Evaluation of concentrations of viral RNA, NSP, web protein (webP) and host factor (host) in subdomains of the spatio-temporal resolved volume model which is based on a tetrahedral volume grid with the exclusion of the ER lumen volume.

Our framework allows the evaluation of the concentrations at each point in space (of the domain) and time. Figure 12 shows the evolution of the integration of the concentrations of the ingredients within different subdomains (exemplaric values shown, evaluation possible for all ingredients at all spatital points and/or subdomains at any time point).

## 4. Discussion

The present state of the art with regards to HCV-specific computational virology is that models of HCV replication are represented by means of homogenized ODE models which are capable of describing a lot of important effects, cf., e.g., [5,6,7,8,9,10,11,12,13,14,15,16,17,18].

However the spatial component of the dynamics plays an important role to develop a complete and well-founded understanding of HCV replication dynamics. For example, it would be important to understand why NS5A gets redistributed and changes mobility following application of NS5A inhibitors [32], and it would be interesting to understand the role of the spatially non-homogeneous and discontinuous distributed membanous web regions [30].

Therefore, we are developing spatially resolved models of the HCV vRNA replication cycle [1]. In this paper, we presented the development of spatio-temporal resolved models of the vRNA replication cycle and corresponding simulations of our models. Our approaches mimic the interplay of vRNA, non structural proteins/membranous web growth and a host factor for directing the vRNA reproduction cycle in a spatio-temporal resolved manner. Our PDE models are highly related to the realistic reconstructed ER geometry (either its surface or to the cytosol as established by means of the ER surface forming its boundary, depending on the model) acknowledging the major role of the ER in plus strand RNA virus replication [3].

Indeed, the equations we use are in principle just the extension of the state-of-the-art standard HCV ODE models which describe all processes with space-independent reaction constants, cf., e.g., [5,6,17,18], even so so far with less states. Our extension to the spatial description however gets performed from the very beginning upon highly realistic geometric environments.

Therefore, our simulations already by now capture the main events of the vRNA replication cycle as we have shown with the simulations as presented afore in Section 3:

When a HCV virus particle enters a hepatoma cell, it uncoates its vRNA. The vRNA moves to the ribosomes. Nonstructural viral proteins get produced, create the webs where the vRNA gets replicated. The vRNA moves again to ribosomes, the vRNA cycle is closed and starts again respectively continues. Our simulations based upon the models as developed within Section 2 and presented within Section 3 reproduce these events based on different mathematical descriptions which leads to biologically meaningful simulations. Nevertheless, our approach is so far more qualitative and also restricted to a specific sub-part of the ER.

We created two types of models: Models which focus upon the restriction of the movement of viral components to the surfaces of the ER and the webs (motivated by the experimentally well-founded anchoring of the NSPs to the ER surface) and a model which allows for the movement of all components within the complete cytosol and and the web regions with the exclusion of the ER lumen (motivated by the assumption that the host factor and maybe also vRNA movement is not restricted to any surface but take place within the volume of cytosol and webs.)

Indeed, these approaches presumably have to be combined in the middle run in subsequent work. Nevertheless, we consider them to be a first but well-founded approach of spatially resolved HCV models.

Let us evaluate the models critically in detail:

### 4.1. The Suface PDE Models

Our sPDE models represent the first approach of surface bound dynamic descriptions of the major components of the vRNA replication cycle.

We presented two types of this model type: One model incorporated the polyprotein state while the other model neglected this intermediate step due to the time scale which is short in comparison to the other events of the process.

While the corresponding simulations reproduce the main effects of the vRNA cycle, the reproduction process of the vRNA is not restricted to the web surface in reality, but moreover takes place within the volume of the webs. Nevertheless we restrict the vRNA reproduction to the surface of the web regions in this model. The latter fact is of minor importance for the quality of the surface models at this model stage since the surface of the webs and their volume are comparably at similar scales within the geometric structure we analyze, thus we may use surface descriptions of the replication processes within the surface PDE based models. The same argument applies to the related dynamics of the growth of the membranous web regions by means of web protein accumulation. However, a minor disadvantage of the surface models presented here is that vRNA movement is restricted to the ER surface even though experimentally it is not clear how vRNA moves (cf., also the discussion in this paper in Section 4.6). Eventually, the vRNA may move also in the cytosol. In particular, it is doubtful if the host factor movement should be restricted to the surfaces of ER and the web regions. Therefore, we developed also a model where all components move within the complete volume of cytosol and web regions while the ER lumen is excluded from the computational domain.

### 4.2. The “Volume” PDE Model

In the new volume model, the movement of the nonstructural viral proteins, vRNA and the host factor is possible within the complete cytosol volume (with the exclusion of the ER lumen as indicated also experimentally, i.e., the ER lumen does not participate in the vRNA cycle as indicated also by experiment [1]) and the volumes of the membranous web regions. However, this model neglects the important biophysical property to restrict NSP movement to the ER surface which was clearly proved by experiment [1], in contrast the movement of the viral proteins is possible within the complete cytosol. Therefore, we developed the afore mentioned models which take into account the attachment of the viral proteins to the ER surface.

### 4.3. Comparison of Our PDE Models to ODE Models—Relationship Form/Function

To the best of our knowledge and understanding, a basic ODE model of HCV replication kinetics at the cellular level has been presented in [6], whereas an extension of this model has been recently proposed, for example, in [17]. These models enable a description of the overall kinetics of the basic compartments of HCV replication. Dahari et.al. [6] introduced an ODE model describing the interaction of various compartments which are separated into cytosolic and web bound parts. Basic components are RNA, translation complex, polyprotein, NSP polymerase and different states of double stranded RNA, which arise from multilinear reactions of the other constituents. The core of the model is given by the multilinear transition and production rates of RNA which is resolved by means of various intermediate steps (namely the transition rates of the constituents between the the cytosol and the webs and the afore mentioned various intermediate states of the RNA). The RNA production is based on a detailed description of different forms of intermediate replication complexes that appear in several places of the model equations in the form of multilinear reactions of NSP polymerase and web bound RNA. The latter ones form other states of replication complexes with the same characteristics, and incorporating double strand RNA. Another important feature of the RNA kinetics model presented in [6] is that there is one unknown, corresponding to one type of RNA and NSP concentration, for each compartment *and* for each aggregation state in which the constituents appear.

In part, some of the states of the RNA which intend to mimic spatial states of the RNA within the ODE models are entire part of one RNA state at different spatial locations. In our spatial model, this distinction appears more natural. Nevertheless, some of the biophysical bound states still could be resolved more finely also within our approach because in some cases, the distinction just based upon the spatial location is a rough first approach and it will be useful to introduce different states at the same spatial location, based upon different biophysical states.

The reaction terms describing the evolution of the different aggregation states of the double strand RNA are multilinear functions. These functions describe the production of new (web bound) RNA. The newly synthesized RNA forms in part new replication complexes and in part reacts into cytosolic free RNA. Degradations of various components are included. To this end, the cell is considered to be homogeneous, and those time delays which arise from transport are modeled by means of reactions which replace transport by transition rates. All in all, the reactions which describe the transitions and productions from and to different states are in various cases effective reactions which incorporate also the transport properties. The model harbored various unknown parameters that were fitted such that the steady state properties of experiment were reproduced. The fitting of the unknown parameters of the afore described multi-parameter model to experimental data as extracted from averaged samples of in vitro experiments allowed for the design of a model which was capable of describing the spatially homogenized dynamics of a sample of cells [6].

Binder et.al extended the model of [6] substantially [17]. The authors of [17] measured the homogenized RNA levels within the first hours after virus infection of the in vitro cell samples. Hence, in [17], an ODE model in a similar style as afore [6] but with various extensions was developed which allowed for the description of the overall dynamics of the RNA levels of the cell samples as well within the first hours after infection and also for the steady state phase. Northern blot analysis of the viral plus- and minus-strand RNA formed the experimental basis of the fit of the model to the data of [17], i.e., all data were averaged over a sample of cells. The principle of spatial homogenization was applied and the homogenized ODE compartment model was able to capture the effects of the homogenized sample to reproduce the dynamics of the system [17]. The authors of [17] also introduced a host factor which was crucial for fitting their model to the dynamics of RNA replication. Therefore, we used the host factor principle of [17] as inspiration to avoid the RNA breakdown which also else would happen within our model framework. Binder at.al [17] also used trilinear reaction terms incorporating the host factor for RNA replication as in principle in our case. Thus, the model of [17] allowed for a distinguishing between high and low permissive in vitro cell lines due to different host factor properties. (A cell “line” is a term of virology to denote a special cell type.)

While the ODE model of [17] established the influence of the host factor, a spatial model could help for resolving the origin of the difference in the host factor influence for e.g., different cell lines which is beyond the potential of compartment models.

The authors of [17] further performed a sensitivity analysis to figure out which reactions of the ODE model have the most impact onto the RNA replication, searching hence for how to break the RNA cycle the most effectively. This analysis gave insight into the sensitivity of the RNA production to various intermediate steps. Since the reaction rates of the ODE models are effective reactions which may incorporate transport phenomena, the PDE based ansatz has the potential to resolve this sensitivity analysis in more detail, for example by disabling some of the webs in the simulation environment or to change diffusion parameters to test for the influence of the interplay of transport and reaction as we have demonstrated afore in this study. Such efforts are much more difficult to perform within experimental approaches compared to our simulation framework.

Degradation effects are so far neglected in our approach due to the proof-of-concept of the qualitative model without fit to experimental values. The introduction of reactive degradations is technically easy within our framework and has to be done once experimental values available.

To this end, the ODE models of [6,17] enabled a lot of insight into various processes of HCV replication which may be considered at a spatially homogenized level.

Hence, the models of [6,17] represent approaches to model various basic effects of HCV replication by means of techniques homogenizing the concentrations over the complete space hence neglecting the spatial component. Such approaches are always useful as long as the spatial aspect may be neglected at a first approach or as long as spatial effects are negligible. This assumption holds true for the questions of [6,17] and their respective answers allowed for deep insight into HCV replication dynamics. However, there remain other questions which are rather difficult to solve upon a pure ODE approach which neglects the spatial aspect.

Indeed, our PDE models are in principle an extension of the ODE models of the [6,17] type to the spatially resolved case for acknowledging those effects which may not be resolved as long as spatial resolution is neglected and for approaching to a higher resolution with respect to the details of the underlying processes.

Therefore, while the models of type [6,17] are already realistic, our model approach may allow for more detailed insight. For example, the step structure of RNA replication which we have shown qualitatively once a new web starts its work has not been proposed upon ODE compartment models. Of course, it will remain to future experiments to investigate if the step-structure will be a main feature of spatial HCV models, or if other aspects will predominate based upon our new approach.

The overall kinetics of our PDE ansatz differs to basic ODE models like e.g., [6,17] in particular in the following way:

The reaction terms are restricted only to the regions of the interior of a cell in which it is known from biology that they are allowed to occur.

Also in the limit case of no diffusion, the spatial resolution of the reaction terms implies that the kinetics of the considered problem is not space-independent. Rather, different reactions occur in different places and, in principle, also non simultaneously. In a model based solely on ODEs, these aspects of the phenomenology cannot be resolved, and the reaction terms should thus be understood as effective. The fact that the concentration of an agent, e.g., the concentration of the viral RNA, *R*, features in different cellular regions (indeed, *R* appears both in the reaction term defined over the web-regions and in that defined over the ribosomes) means that the considered substance has to “travel” from one region to the other in order for the reactions to take place. Moreover, this travel (in fact, a transport process) is characterised by time-scales that are generally different from those associated with the reaction terms and by length-scales that require a fully spatial resolution of the kinetics. This is the main reason for justifying the introduction of diffusion in the model equations.

In particular, as stated afore, ODE approaches allow comparisons to the averaged sample of various cells evaluated together, but they do not allow, for example, detailed comparisons to spatially resolved fluorescence data where the processes are considered in detail. To understand spatially resolved observations like fluorescence microscopy data rather then e.g., Western/Northern blot analyses of samples of cells, spatially resolved models are unpreventable .

Therefore, we cite [17] concerning the webs: *“The exact architecture and topology of these structures, and particularly their structure-function-relationship, is not fully understood yet”*. It seems to be rather unlikely that an ODE model bears the potential to resolve for example this important question of the relation of form and function. However, PDE models bear the potential to resolve the relationship of form and function as we have demonstrated in [53] for the case of presynaptic boutons within computational neuroscience. A PDE model could help for resolving, e.g., if the difference in the host factor for different cell types arises from its entire initial concentration, or from the transport properties. Our investigations as shown in Section 3.1.1 and Section 3.1.2 suggest that the availability of host factor in a cell line may not only depend upon its total amount within the complete cell, but moreover upon the transport mechanisms offered by the cell line.

Finally, also the switching-off of single webs is rather difficult to simulate within ODE models, while it appears natural within PDE models at realistic reconstructed geometries.

The overall behaviour as depicted in Section 3.1.1 and investigated with respect to parameter variations in Section 3.1.2 demonstrated an important impact of the RNA and host factor transport properties upon the global kinetics. In particular, the transport of the RNA to the next webs caused a step-like influence upon the RNA uprise kinetics. These properties have to be investigated in more detail by means of adequate new experiments, based, e.g., upon fluorescence data of single cells with high spatial and temporal resolution. If the spatial propagation of RNA will be revealed by experiments to be different from the one suggested by us, our model will need further refinement, since such a different behaviour may cause different kinetic properties. In any case, the overall kinetics of the dynamics of the levels of the concentrations within single cells presumably may have not so continuous structures as they appear within evaluations which are homogenized over the space.

To this end, our spatial framework creates, for example, a basis for the detailed investigation of the relation of form and function during HCV replication within single cells. Our virus model of this study is a first step on this way for virology purposes to resolve, e.g., questions of form and function, which of course still will need ask for a lot of forthcoming efforts on both sides, in silico *and* in vitro/in vivo.

### 4.4. Critical Summary and Potentials of Our Models

We have developed new fully 3D spatio-temporal resolved models of the HCV vRNA replication cycle. Intriguingly, these models are capable of reproducing the main events of the vRNA cycle (as known from experimental analysis) in a qualitative but spatio-temporal resolved manner. Our simulations demonstrated the potential of new directions for research, which we initiate by means of the spatial framework we introduce for mathematical modeling of viral replication cycles. Therefore, we predict that the introduction of spatial resolution could represent a significant step forward for approaches to computational virology in the future.

We present a model of HCV vRNA replication dynamics based upon a realistic reconstructed geometric environment of an immonustained hepatocyte, i.e., we developed and simulated a 3D spatio-temporal resolving PDE model mimicking the HCV vRNA replication cycle within a specific subsection of a realistic geometry based on experimentally-derived data. The parameters of the diffusion-reaction PDEs we use so far are heuristic, however it is important to fit the parameters from new experimental data.

The model is capable of covering all major steps of the vRNA replication cycle dynamics in a spatially resolved manner: vRNA uncoates, translates viral proteins, the viral proteins cause the growth of the membranous web, the vRNA gets replicated inside the membranous web. The newly synthesized vRNA moves again to the ribosomes where new NSPs are produced. Thus, the first vRNA cycle is complete and then subsequent cycles continue. During vRNA reproduction, a generic host factor gets consumed which aims the involvement of the host into the vRNA polymerization and which prevents unlimited vRNA reproduction (which would not be biophysically sound).

The state-of-the-art ODE model computations effectively perform summations of each component (like vRNA or NSPs) over the complete cell. Our simulations allow for the evaluation of the concentrations of the components of our models at each point in time and space of the computational domain. Our simulations are visualized as movies and hence allow the observation of the in silico processes in a manner similar to observations of in vivo/in vitro experiments by microscopes, however with the advantage of a much higher resolution, though with some obvious restrictions, such as:

At first, the location of the ribosomes is simplified in both approaches presented here, also due to a lack of exact experimental information. Presumably, it would be comparably easy to get experimental data concerning this aspect.

Further, it does not seem to be very realistic that the vRNA may diffuse away while it gets translated and may get, in part at the same local point, even replicated.

Therefore, to make the model more realistic, it will get necessary to distinguish between different states of the components, e.g., to treat vRNA different when it is bound to ribosomes compared to when it gets replicated inside the webs.

Also, the multilinearity of the reproduction process of the vRNA, cf., Equations (2a)–(2d) (the surface PDE model and all subsequent simplifications of this type) resp. Equations (13a)–(13d) (the volume PDE model), is valid only for small concentrations. However, we use this type of reaction since it is also state of the art of the ODE models.

Further, the coupling of surface based events and volume based events, i.e., the coupling of 3D sPDEs and 3D volume PDEs will become important, since some agents like the NSPs are bound to the ER surface, however other agents like the host factor are presumably able to move in the cytosol, the web proteins are able to move within the volume of the web regions, i.e., within a volumetric framework. Thus, in future research, forthcoming models will need to combine volume effects like host factor diffusion, and surface effects as they are proven experimentally for the NSPs (as they are realized within the afore explained surface models). Such couplings of sPDEs with volume PDEs will cause further challenges referring to modeling but also to numerical evaluation techniques.

Finally, one will also have to take into account that even though actually we call the limiting factor “host factor”, that host factor may play a different role in this context, it could even get enriched in the web regions as it was recently shown for ATP [3].

Within future research, strong interest could refer to the question whether there any general characteristics of the ER and the web structures that can be used to generate random structures. For example, it would be interesting to evaluate the average distance between webs and the average web volume. Also the curvature structure of the ER surface and the ratio of e.g., volume to surface or the ratio of length of ER branches to volume could be of interest. Such a question could be handled with the help of e.g., SpineLab [54] which allows for the generation of characteristic skeletons. Also we have various tools which we apply already sucesfully within spatially resolved computational neuroscience like NeuGen [55] which could serve as basis also for creation of artificial ERs. Especially when trying to apply this modeling framework to novel experimental data it would be interesting to know if there are some specific characteristics that determine the topology of the ER and membraneous web.

To date, our work focused more upon the development of a model using geometric structures derived from real experimental data rather than artificially created geometries. In any case, the question gives rise for interesting future studies which are beyond the scope of the present paper.

The present study represents the first step of the development of a model that takes into account the geometry of the ER. To derive results which can be verified experimentally, realistic geometries are needed. Therefore, these studies could serve as basis for the identification of the crucial parameters of the geometric structure and hence serve for the randomly based creation of artificial but reality-similar geometries. This is one of the reasons why we perform 3D studies—to investigate for the influence of the geometry upon the dynamics. Therefore, future work will have to incorporate the comparison of the results of the dynamics for different realistic reconstructed cell environments. Such work we already performed for the estimation of the NS5A diffusion constant based upon experimental FRAP time series cross combined with different topologies of ERs to investigate the influence of the geometry upon the result [40]. Our results (which are so far unpublished) demonstrate clearly the strong influence of the geometry upon the process dynamics of even one single ingredient of the vRNA cycle—depending on the geometric structure, the transport “velocity” of the NS5A spread changes strongly. The propagation velocity of NS5A over the cell depends strongly upon the ER itself, and hence also the vRNA cycle itself will change its dynamics depending on the structure of the ER.

During the way to make our model quantitative, we will include of course such checks and quantitative validations with different geometries into the project. The aim of this paper is to introduce the model framework itself and to demonstrate its potential in a qualitative manner.

To this end, our approach is based on two columns which are intended to grow together in the middle run: On the one hand, we are doing parameter estimations of single components of viral replication based on the Gauss-Newton algorithm in order for extracting e.g., the diffusion constant of basic viral proteins on the surface of the ER using experimental FRAP (fluorescence recovery after photobleaching) time series [37,40,56].

On the other hand side, we are developing (as presented here) the model which mimics the interplay of all important components of virus replication, e.g., viral RNA, nonstructural viral proteins and a host factor. The estimated parameters are entering the model step by step. Therefore, the presented work is a practical application of the powerful simulation software tool UG4 [34,36] and its multigrid techniques in the context of modern biophysical research. The computations are performed at leading supercomputers [37] and hence bear the potential of fast and efficient comparisons to experiment once the model gets quantitative.

### 4.5. Multiscale Modeling

Even though the analysis of small fractions of a cell could help for new helpful insights, we suggest that for a complete understanding, complete cells will be needed to derive a complete understanding. We suggest the extension of the model to complete cells will have to go hand in hand with the adaption of some parameters which could be based in part upon small fractions of experimental cells. However, we suggest small fractions may have special behaviour which changes too strongly from one region to the other, indicating that consideration of complete cells will become necessary in the long run. Of course, some questions could be answered by considering only small fractions of cells, while other questions will likely need to account for the whole cell.

As next steps, there are two project directions in our view: On the one hand side, we intend to extend our model from small pieces of the ER to the complete ER. The computational domain needs to be extended to complete cells once experimental data are available which allow for a fitting of all parameters of the basic PDEs. Complete ERs were already reconstructed by us for various hepatocytes represented by fluorescence z-stacks, cf. for more detail [40]. On this way, a coupling of surface PDEs to take into account surface effects and “volume” PDEs for volume effects will get necessary for sure.

On the other hand side, the model has to get realistic quantitative parameters. In particular, we intend to use realistic parameters as far as ever possible, like e.g., the afore mentioned estimated (so far unpublished) NS5A diffusion constant.

In the other direction of scale, a future aim could be to make a more detailed model of spatially resolved webs, i.e., a single web based e.g. on electrotomography data [2] taking into account the different vesicle types as DMV or Single or Multi Membrane vesicles (SMV and MMV) [2].

Future models could incorporate vRNA and protein degradation. The inclusion of degradation effects is trivial within UG4, therefore we omitted this small point within our first approach.

An important milestone of the model development will be to be able to reproduce quantitative data such as [38,39].

Besides all restrictions as discussed above, our approaches show the potential of spatial models of virus replication at realistic reconstructed geometric structures. Since many details are still unclear even qualitatively also in experiment, our approach represents a good basis for extensions in the interplay of model extensions and new experiments.

### 4.6. The vRNA Transport and the Web Movement

In particular, further improvements will ask for the afore mentioned improvement of the vRNA movement. A delicate detail will be the question if indeed the vRNA “cloud” diffuses just around, causing the vRNA/NSP front to move like a wave through the ER; or if the RNA moves around and binds in a stochastic manner, causing not a wave-like spread of the webs, but moreover a spread in the way as if “lights” would be switched on randomly in a random order randomly distributed in time and space all over the ER, until the ER is filled completely. This could be a point which could be tested also experimentally and would have strong impact upon our forthcoming model improvement.

Of big interest and challenge will be the aim to model the movement of the small web particles as described at first by [30]. The modeling/simulating of the web movements will get a technical challenge, as well with respect to the physical description on the one hand side as well as the technical realization within the FV context. Insights could be gained by experiments which we propose for this sake also in order for getting a basic idea of how the particles move spatially.

### 4.7. Future Perspectives of Spatial Virus Modeling

In the medium term, our model development has the potential to help contribute to a more detailed understanding of the viral replication dynamics, hence allowing for the planning of more straightforward experiments.

In the course of the afore mentioned possible model improvements, we suggest that our framework may allow for substantial input for experimental design in order for augmenting the “mechanistic”, i.e., mathematical-biophysical, understanding of the HCV replication dynamics, also in order of having a tool to give support in the context of the development of new DAAs.

In the long run, our model development has the potential to give substantial impact on the quantitative understanding of those basic virus replication properties which are dependent strongly upon spatial patterns, like, e.g., the interplay of form and function of intracellular virus replication mechanisms. Therefore, our approach may be considered as an entire interdisciplinary pathway with substantial future perspective.

### 4.8. Context of ER Related Research

Only few approaches so far take into account realistic ER geometries in the literature to our best knowledge at all in any biological fields. Indeed, we only could find [57] which calculate Calcium dynamics on top of a reconstructed ER geometry based on electrotomography stacks using Finite Element (FE) methods and a tetrahedral grid representing the ER lumen. This approach remembers us to our method shown in Section 2.2 and Section 3.3.

Anyhow, the ER takes a major role within various fields of pathogenesis. For example, the authors of [58] have shown recently the important role of the ER respectively its disfunction within the pathogenesis of Alzheimer. Therefore, also in such a context, spatial models could help for new insights.

To this end, our new approach opens new gates of basic research for various fields of medicine and biophysics and even for clinical applications in the long run, not only, but in particular also, for virus infections.

## 5. Conclusions

We presented spatially resolved models of the HCV vRNA replication cycle evaluated using a realistic reconstructed cell micro-environment involving the ER and the membranous web regions. To our best knowledge, this paper represents the first spatially resolved approach to describe the dynamics of any vRNA cycle at the (intra)cellular level.

We demonstrated that spatially resolved dynamic models of virus replication within realistic geometric environments harbor substantial potential to overcome various present limitations of the understanding of virus replication properties, concerning, e.g., the relation of form and function. We demonstrated that the transport based mechanisms within the complex geometric environment of ER and membranous webs caused characteristic shapes of the overall kinetics of, e.g., the intracellular RNA levels within single cells, suggesting the amount of the major concentrations of the vRNA cycle may be strongly related to the geometric structures, i.e., form and function may influence each other mutually in a substantial manner.

The simulations motivate new experiments and intensive theoretical-experimental interdisciplinary approaches such as to reveal why the application of NS5A inhibitors causes a spatial redistribution of NS5A and a motility change, accompanied by an immediate breakdown of the vRNA replication cycle [33]. Namely, we expect the need for, e.g., detailed fluorescence experiments at single cells with high temporal and spatial resolution.

The proposed spatially resolved models may also allow extensions to other virus infections, e.g., the Dengue virus [59,60] as another Flavivirus [3], or even other viral families (like Filoviruses (e.g., Ebola and Marburg) or retroviruses (e.g., HIV)).

## Figures and Tables

**Table 1:** Geometry info of the 3D surface ER representation and the sPDE model of 4 concentrations Equations (2a)–(2d). DoF number at base level and one refinement.

Type	Number
vertices	5645
edges	17,124
faces (base)	11,467
faces (1 ref)	45,868
volumes	0
DoF (base)	22,580
DoF (1 ref)	91,076

**Table 2:** Basic parameter set for the sPDE model evaluations. We emphasize that our code is not restricted to these parameters in any way and the stability does not depend on the use of the parameters reported in the table. Note: Web number parameter given by microscopy data.

Parameters	Value	Unit
$D_{R}$	0.3	$\frac{{\left(\mu m\right)}^{2}}{s}$
$D_{P}$	0.3	$\frac{{\left(\mu m\right)}^{2}}{s}$
$D_{W}$	0.5	$\frac{{\left(\mu m\right)}^{2}}{s}$
$D_{H}$	0.05	$\frac{{\left(\mu m\right)}^{2}}{s}$
$r_{1}$	15	$s^{-1}$
$r_{2}$	100	$s^{-1}$
$r_{3}$	90	$s^{-1}$
$r_{4}$	1.5	$s^{-1}$
$R_{0}$	10	$\frac{1}{{\left(\mu m\right)}^{2}}$
webs	7	#

**Table 3:** Geometry info of the 3D continuum ER representation which serves as basis for the PDE model Equations (10a)–(10d). DoF number at base level and with one refinement.

Type	Number
vertices	16,331
edges	104,282
faces	169,565
volumes	81,619
DoFs (base)	65,324
DoFs (1 ref)	482,452

**Table 4:** Basic parameter set for the vPDE model evaluations.

Parameters	Value	Unit
$D_{R}$	1	$\frac{{\left(\mu m\right)}^{2}}{s}$
$D_{N}$	3	$\frac{{\left(\mu m\right)}^{2}}{s}$
$D_{W}$	5	$\frac{{\left(\mu m\right)}^{2}}{s}$
$D_{H}$	0.1	$\frac{{\left(\mu m\right)}^{2}}{s}$
$r_{1}$	0.2	$s^{-1}$
$r_{2}$	2	$s^{-1}$
$r_{3}$	20	$s^{-1}$
$r_{4}$	0.067	$s^{-1}$
$R_{0}$	10	$\frac{1}{{\left(\mu m\right)}^{3}}$
$H_{0}$	1	$\frac{1}{{\left(\mu m\right)}^{3}}$
webs	7	#

## Supplementary Materials

The following are available online at www.mdpi.com/1999-4915/9/10/282/s1, Supplemental Movie S1 Video: 3D surface PDE model, 3 concentrations (vRNA, webProtein, host factor), rotating view, Supplemental Movie S2 Video: 3D surface PDE model 4 concentrations (vRNA, polyprotein, webProtein, host factor), static view, and Supplemental Movie S3 Video: 3D “volume” PDE model. (cf. also the Appendices Appendix A, Appendix B and Appendix C for brief descriptions of the simulation movies. Extended descriptions can be found in the main part of this paper, cf. Section 3.)

## Abbreviations

The following abbreviations are used in this manuscript:

HCV	Hepatitis C virus
vRNA	viral RNA
NSP	non structural viral protein
SP	structural protein
ODE	Ordinary Differential Equation
PDE	Partial Differential Equation
sPDE	surface Partial Differential Equation
vPDE	(“volume”) Partial Differential Equation
FD	Finite Differences
FV	Finite Elements
FV	Finite Volumes
MG	Multi Grid
GMG	Geometric Multi Grid
UG4	Unstructured Grids version 4 [34,36]
NeuRA2	Neuron reconstruction algorithm, version 2.3 [40,43]

## Appendix A. S1 Video: 3D Surface PDE Model, 3 Concentrations (vRNA, webProtein, Host Factor), Rotating View

The evaluation of the simple and simplified sPDE model: vRNA, webP and host factor. Rotating perspectives, concentrations dynamics on ER surface shown from different rotating perspectives. ER cut view, initially perspective from behind, later rotating to front perspective. Short description (for a much more detailed description of the events, cf. Section 3.1): We see how the initial vRNA translates at the ribosomes directly web protein which diffuses into the predefined web region. During the rotation process (which is just for presentation reasons) on sees from the front how the webs grow into the experimentally predefined web regions. The web protein only may diffuse into the web region directly located to the ribosomes where it got produced. The vRNA diffuses as well into the growing web region and also on the ER surface where it diffuses away. Within the web region, the combination of vRNA and bound web protein produces new vRNA consuming on this way the host factor which gets support from diffusing host factor from outside. (A host factor is introduced to avoid unrealistic production explosions which else would appear). Since the vRNA gets reproduced in the web region proportional to the web protein concentration, the vRNA gets augmented in the web region(s) and hence a significantly enhanced amount of vRNA will spread starting from the web. The newly produced vRNA diffuses to the ER surface and from there it diffuses to other predefined intersection regions of ribosomes. There, it translates web protein which may only diffuse into the directly connected web regions to copy again the vRNA. In this way, the ER surface gets filled with vRNA and the webs get filled with web protein and vRNA. During the vRNA reproduction process, the host factor gets consumed. Once the host factor is consumed, the corresponding web gets ineffective and the vRNA replication stops. Since the vRNA reproduction is also proportional to host factor, a breakdown of vRNA or NSP production cannot take place.

## Appendix B. S2 Video: 3D Surface PDE Model 4 Concentrations (vRNA, Polyprotein, webProtein, Host Factor), Static View

The evaluation of the 4 agents sPDE model: vRNA, polyprotein, webProtein and host factor. View static, concentrations dynamics on ER surface shown from different but fixed perspectives. The perspectives are in some sectors from the front and in others from behind. In some of the sectors, the ER is opened by means of a cut plane, but not in all. Short description (for a much more detailed description of the events, cf. Section 3.2): The version of the model incorporating and not neglecting the intermediate NSP production aiming the polyprotein is also evaluated for completeness and for introducing a static simulation movie introduction version. The process described by these sPDEs read in biological language as follows: vRNA is located at one ribosomal region at the beginning of the process, the vRNA translates viral polyproteins at the ribosomes. The polyprotein cleaves fast into the web protein which forms the web directly within the predefined membranous web region. The polyprotein only may diffuse within the ribosomal region and the web protein only may diffuse in the ribosomal and predefined web region, i.e., the experimental web region. The host factor gets consumed in parallel when the vRNA gets replicated within the webs. Indeed, such a host factor is very realistic because virus replication depends on various host factors, once they are consumed, virus procedures cannot continue locally. Inside the webs the vRNA gets replicated, and diffuses away, in particular also to ribosomes for new polyprotein creation, and so on. Anyhow the intermediate polyprotein state does not have an important influence within this model step, since it gets transformed effectively directly into web protein after translation. Therefore, it is justified to approximate this model with the effective exclusion of this intermediate step.

## Appendix C. S3 Video: 3D “Volume” PDE Model

The evaluation of the 3D PDE continuum model in the cytosol with the exclusion of the ER lumen. vRNA, NSP, web protein (WebP) and Host factor. Concentration dynamics displayed by means of a cut plane of the volume grid. Parameters of the PDE heuristic. For a detailed description, cf. Section 3.3.
